# Neural correlates of static and dynamic social decision‐making in real‐time sibling interactions

**DOI:** 10.1002/hbm.26788

**Published:** 2024-07-19

**Authors:** Lucia Hernandez‐Pena, Julia Koch, Edda Bilek, Julia Schräder, Andreas Meyer‐Lindenberg, Rebecca Waller, Ute Habel, Rik Sijben, Lisa Wagels

**Affiliations:** ^1^ Department of Psychiatry, Psychotherapy and Psychosomatics Faculty of Medicine, RWTH Aachen Aachen Germany; ^2^ JARA ‐ Translational Brain Medicine Aachen Germany; ^3^ Wellcome Centre for Human Neuroimaging, Institute of Neurology University College London London UK; ^4^ Department of Psychiatry and Psychotherapy, Medical Faculty Mannheim, Central Institute of Mental Health Heidelberg University Mannheim Germany; ^5^ Department of Psychology University of Pennsylvania Philadelphia Pennsylvania USA; ^6^ Institute of Neuroscience and Medicine JARA‐Institute Brain Structure Function Relationship (INM 10), Research Center Jülich Jülich Germany; ^7^ Brain Imaging Facility, Interdisciplinary Center for Clinical Research (IZKF) RWTH Aachen University Aachen Germany

**Keywords:** fMRI, hyperscanning, mentalizing, siblings, social decision‐making, social interaction, theory of mind

## Abstract

In traditional game theory tasks, social decision‐making is centered on the prediction of the intentions (i.e., mentalizing) of strangers or manipulated responses. In contrast, real‐life scenarios often involve familiar individuals in dynamic environments. Further research is needed to explore neural correlates of social decision‐making with changes in the available information and environmental settings. This study collected fMRI hyperscanning data (*N* = 100, 46 same‐sex pairs were analyzed) to investigate sibling pairs engaging in an iterated Chicken Game task within a competitive context, including two decision‐making phases. In the static phase, participants chose between turning (cooperate) and continuing (defect) in a fixed time window. Participants could estimate the probability of different events based on their priors (previous outcomes and representation of other's intentions) and report their decision plan. The dynamic phase mirrored real‐world interactions in which information is continuously changing (replicated within a virtual environment). Individuals had to simultaneously update their beliefs, monitor the actions of the other, and adjust their decisions. Our findings revealed substantial choice consistency between the two phases and evidence for shared neural correlates in mentalizing‐related brain regions, including the prefrontal cortex, temporoparietal junction (TPJ), and precuneus. Specific neural correlates were associated with each phase; increased activation of areas associated with action planning and outcome evaluation were found in the static compared with the dynamic phase. Using the opposite contrast, dynamic decision‐making showed higher activation in regions related to predicting and monitoring other's actions, including the anterior cingulate cortex and insula. Cooperation (turning), compared with defection (continuing), showed increased activation in mentalizing‐related regions only in the static phase, while defection, relative to cooperation, exhibited higher activation in areas associated with conflict monitoring and risk processing in the dynamic phase. Men were less cooperative and had greater TPJ activation. Sibling competitive relationship did not predict competitive behavior but showed a tendency to predict brain activity during dynamic decision‐making. Only individual brain activation results are included here, and no interbrain analyses are reported. These neural correlates emphasize the significance of considering varying levels of information available and environmental settings when delving into the intricacies of mentalizing during social decision‐making among familiar individuals.

## Graphical abstract

Using the Interactive Chicken Game task, which includes two decision‐making phases (static and dynamic), we measured 100 participants (50 interactive same‐sex sibling pairs). We found that both decision‐making phases activated brain regions related to predicting other's intentions, but the dynamic and more interactive decision‐making phase required stronger mentalizing brain activation.

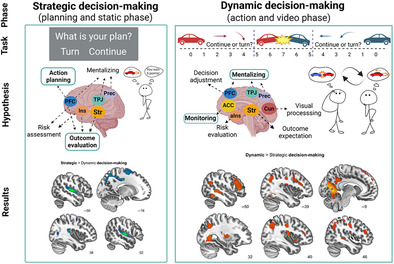

**Practitioners Points**

- A more interactive and realistic version of the Chicken Game task reveals distinct behavioral and neural patterns underlying cooperation and competition depending on the decision‐making phase in real‐time sibling interactions.
- Dynamic decision‐making (continuous belief updating) requires increased activation in mentalizing‐related regions compared with static decision‐making.
- Men exhibit less supportive sibling relationships but show greater activation in mentalizing‐related brain areas during the decision‐making process.

## 1 INTRODUCTION

In the last decade, there has been a strong call for “second‐person” neuroscience, which implies focusing on an interactor's perspective to understand social cognition (Redcay & Schilbach, 2019; Schilbach, 2014; Schilbach et al., 2013). Hence, we need more ecologically valid and interactive tasks imitating real‐life encounters and ongoing social exchange to explore neural correlates of real‐time reciprocal social interactions (Schilbach, 2014). Social interactive contexts require thoughtful decision‐making (Rilling et al., 2008), which can often prove challenging (Macoveanu et al., 2016). Cooperation is a behavior that typically confers greater rewards for all parties involved compared with alternative behaviors. However, selfishness can sometimes benefit an individual more than mutual cooperation (Macoveanu et al., 2016). Individuals can promote or hamper positive outcomes for themselves and others (Decety et al., 2004) by considering their own preferences and goals alongside the responses and strategies of their partner (Sanfey, 2007). Although cooperation and competition have historically been studied separately, or as a binary choice, they exist along a continuum (Bone et al., 2016; Pisauro et al., 2022). Only a few studies have included a continuous design (Hernandez‐Pena et al., 2023; Killingback et al., 1999; Pisauro et al., 2022; Roberts & Renwick, 2003), which more closely resembles real‐life decision‐making and can better capture degrees of cooperation (Hernandez‐Pena et al., 2023; Pisauro et al., 2022).

The highest level of interactivity in decision‐making tasks emerges when the outcomes of participants' decisions are linked to another's decision. This requires high levels of mentalizing (Rusch et al., 2020), involving anticipation of the behavior of another (Frith & Singer, 2008). Mentalizing (i.e., theory of mind) is the capacity to attribute mental states, such as intentions, beliefs, or desires, to others (Frith & Frith, 2006). To make predictions about others' behavior, individuals consider their past behavior, their psychological and social characteristics, and their intentions (Lee & Harris, 2013).

Social decision‐making during strategic games, in general, has been linked to activation in the medial prefrontal cortex (mPFC), temporoparietal junction (TPJ), superior temporal sulcus (STS), anterior cingulate cortex (ACC), striatum, anterior insula, amygdala, orbitofrontal cortex (OFC), and thalamus (Báez‐Mendoza et al., 2021; Lee & Harris, 2013; Schurz et al., 2014; Van Hoorn et al., 2019). Some of these regions form the mentalizing network, including the bilateral TPJ, mPFC, STS, and precuneus (Molenberghs et al., 2016; Schurz et al., 2014; Van Overwalle, 2009). Men, compared with women, showed significantly higher activation during mentalizing tasks in TPJ (Gao et al., 2019), medial frontal regions and thalamus during social decision‐making (Krach et al., 2009), and posterior parietal cortex, bilateral TPJ, and precuneus during appraisals of others (Veroude et al., 2016). More broadly, cooperative versus competitive decision‐making was linked to increased activation in the insula, posterior cingulate cortex (PCC), anterior frontal cortex, medial OFC, superior parietal cortices (Decety et al., 2004), and calcarine sulcus (Thompson et al., 2021; though also see Tsoi et al., 2016). Competitive versus cooperative decision‐making was related to increased activation in the superior frontal gyrus (SFG), mPFC (Decety et al., 2004), and anterior PCC (Fukui et al., 2006). The right TPJ and mPFC showed higher activity during interactions with competitive versus cooperative partners, while interactions with cooperative versus competitive partners were associated with higher activation in the forebrain, ACC, striatum, and OFC (Bitsch et al., 2018). During real‐life interactions, the brain updates information based on the other's behavior balancing between cooperative and selfish choices. However, prior studies often preinformed participants whether their interactions would be cooperative or competitive, or used fake opponents. Research is needed to investigate how the brain perceives social interactions in real time, without prior instructions about the cooperative context and with real partners.

Famous social dilemmas within the framework of game theory, in which cooperative or competitive decisions from two individuals determine the outcome (Thielmann et al., 2021), are the Prisoner's dilemma (PD) and the Chicken Game (CG, also known as Hawk‐Dove or Snowdrift game). Although the majority of prior studies have used the PD, the CG is considered more suitable for investigating cooperative and competitive decisions (Butler et al., 2011), more omnipresent in social life (de Heus et al., 2010), and in real social interactions (Kümmerli et al., 2007; Su et al., 2018). In the CG task, two participants drive virtual cars toward each other, and the first person to swerve loses. The game features complex dynamics where participants must make decisions while constantly observing and anticipating those of their partner (Fukui et al., 2006). To our knowledge, the CG task has only been used with a fixed time window for a binary choice (e.g., see de Heus et al., 2010; Fukui et al., 2006; Wang et al., 2017).

Manipulation of the environmental setting in social contexts, implicating changes in the available information, can elicit different social cognition processes. In the context of reciprocal social interactions, mentalizing has been associated with recursive updating of beliefs from repeated observation of the other's behavior (Devaine et al., 2014). However, most decision‐making tasks in previous literature did not require continuous updating of the representation of others' beliefs in real time. A few studies have compared two different tasks of social interactions changing the material and instructions (e.g., Gallagher, 2000; Gobbini, 2007). As of our knowledge, no task has tested whether social decision‐making processes differ in static versus dynamic contexts. Simulating the CG in a virtual environment can improve participant engagement and immersion, and enhance its resemblance to real‐life situations (Byom & Mutlu, 2013). In standard game theory tasks, participants have to decide based on static information. Such a static decision‐making context is expected to be associated with estimating the probability of outcome events based on priors (previous outcome feedback, and representation of other's intentions) and making a strategic decision. These decision‐making subprocesses have been related to activation in the PFC, ACC, insula, striatum, and superior temporal gyrus, among others (Ernst & Paulus, 2005). Real‐life dynamics, however, demand constant updates to expectations and strategies as individuals observe and adapt to their partner's actions (Yoshida et al., 2010). A dynamic setting is likely related to belief updating, adaptation, and reevaluation of expectations and priors, as well as, making a final decision in a rapidly changing dynamic environment (Nassar et al., 2010) in which consequences of one's actions are immediate and potentially critical.

Findings on static versus dynamic social decision‐making are limited. In a negotiation game, dynamic (i.e., changes in each trial) compared with static environments required more mentalizing skills (De Weerd et al., 2022). In an interpersonal competitive game, all conditions required mentalizing skills, but participants updated their representation of the opponent's mental model in the conditions with new information about the opponent's choice (Assaf et al., 2009). Along with greater levels of mentalizing, this elicited higher activation of regions such as bilateral TPJ, temporal poles, mPFC, and midbrain. Other studies exploring neural correlates of belief updating found higher activation in brain areas including bilateral STS, superior/middle frontal gyri, precuneus/PCC, ACC, and intraparietal sulcus (Huber et al., 2015; Kobayashi & Hsu, 2017; O'Reilly et al., 2013). However, these studies manipulated uncertainty and expectancy in relatively simple tasks with nonsocial contexts.

In the CG, the risky, but potentially most rewarding option is to defect (de Heus et al., 2010). Thus, deciding to continue (defect) may engage distinct cognitive processes when comparing static and dynamic contexts. In the static phase, defection can be a result of strategic reasoning considering different options. Whereas in the dynamic phase, choosing to continue may evoke greater uncertainty regarding the partner's actions when the partner does not turn with increasing time. When continuing, increased risk and enhanced monitoring may activate regions such as ACC and anterior insula (Krain et al., 2006; Mohr et al., 2010; Morriss et al., 2019; Wu et al., 2021), compared with the decision to turn.

In addition, prior game theory studies have largely used preprogrammed or randomized responses for the partner (e.g., Fukui et al., 2006; Rilling et al., 2002; Thompson et al., 2021). However, a handful of studies that have compared friends versus strangers reported more cooperative and prosocial behavior by maximizing the outcome of the other among familiar people (Schreuders et al., 2018; Su et al., 2018). To better understand the dynamics of dyadic decision‐making, it is essential to explore natural, real time, and reciprocal interactions with real partners (i.e., without manipulation or randomized responses, and such that the actions of one partner affect the other and vice versa) while considering the relationship between the dyad (Redcay & Schilbach, 2019).

Siblings are an ideal model for studying social interactions between highly familiar individuals (Rogers et al., 2021) due to their unique characteristics in terms of longevity in their relationship (Michalski & Euler, 2007). Sibling closeness was associated with higher mutual cooperation and lower mutual competition in the PD task (Segal & Hershberger, 1999). Characteristics that might affect sibling closeness and their interaction include sex and age difference, with same‐sex pairs (especially sisters) reporting closer relationships (for a comprehensive review, see Jensen et al., 2023). In our previous behavioral study using the Interactive Chicken Game task, brothers showed more competitive and dominant behavior compared with sisters, who more frequently adopted turn‐taking strategies (Hernandez‐Pena et al., 2023). Sibling closeness was also linked to greater brain activation when making nonrisky decisions (Rogers et al., 2018). However, no prior study has explored the neural correlates of cooperative versus competitive decision‐making in siblings, including sex differences.

### 1.1 Aims and hypotheses

The majority of functional magnetic resonance imaging (fMRI) and behavioral data analyses presented in this article were deviations from our preregistration plan, due to our later realization that the preregistered analytic plan was inappropriate for the collected data (i.e., due to the large variance between pairs in the number of conditions as shown in Figure S1). As such all analyses should be viewed as exploratory and not confirmatory. The following hypotheses are based on previous literature.

The current study aimed to investigate neural similarities and differences during different decision‐making processes in close relationships (siblings). First, we used a hyperscanning setup to simultaneously measure brain activity of participants in a competitive dyadic context applying the Interactive Chicken Game task. This paradigm allows the differentiation of two decision phases—static phase during planning period (i.e., individuals indicate their intention with static information about the other) versus dynamic phase during action period (i.e., participants are driving virtual cars toward each other and can choose between turning or continuing driving). During the first phase (static planning period), prior information about the previous outcome (feedback), as well as the mental model of the other's intentions enables to predict the other's decision, estimate the likelihood of possible events, and make a strategic decision. During the second phase (dynamic action period), participants could dynamically update and optimize their mental model of the motivations and intentional states of the other participant. In other words, continuous feedback about the other's decision promotes updating beliefs and reevaluating priors with new information.

**FIGURE 1:**
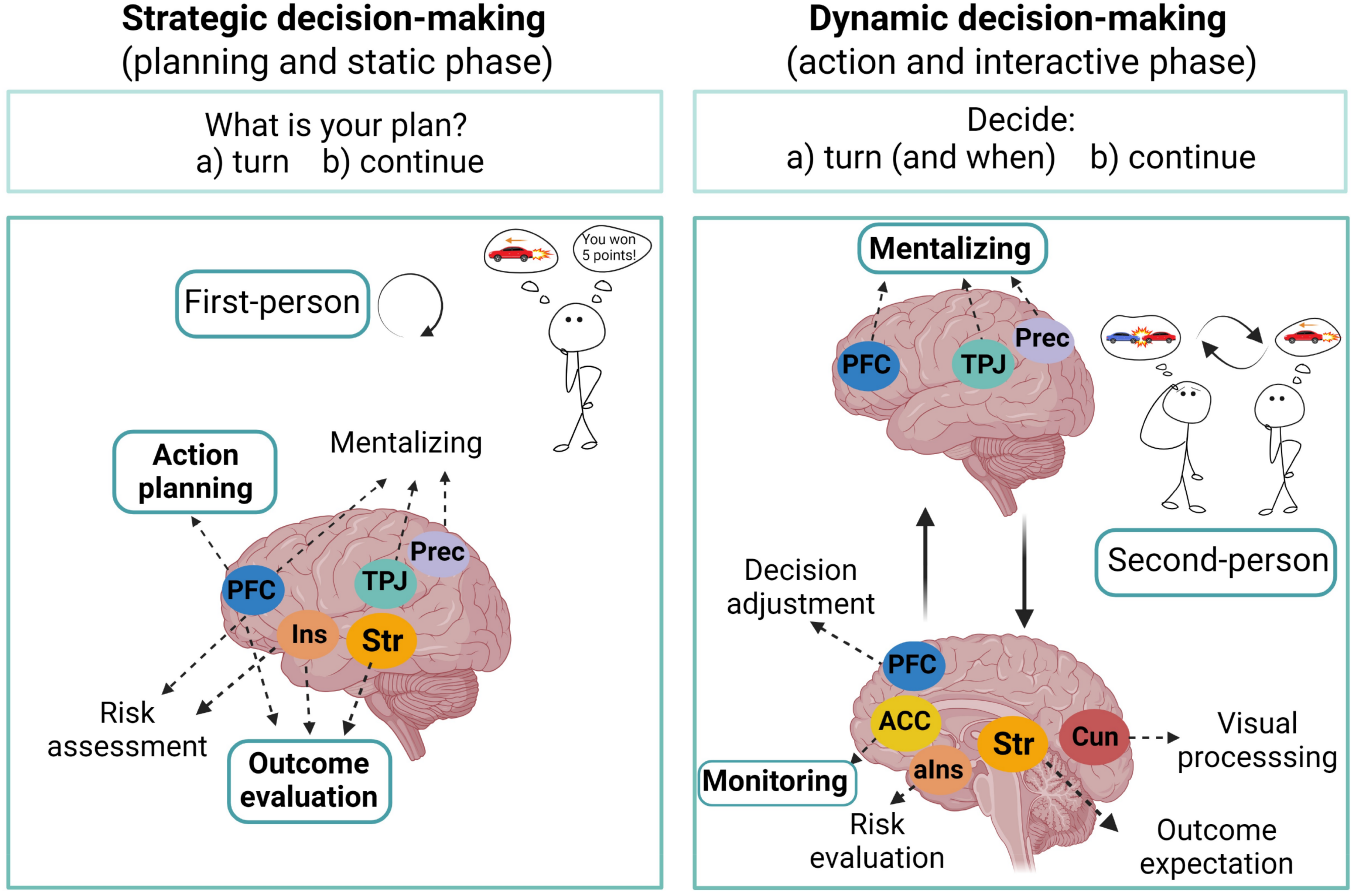
Hypothesized model of cognitive processes and regional brain activations related to each phase of decision‐making. The main cognitive processes in each phase are highlighted in bold and framed. (a) Static (strategic) phase includes the *intention question* “What is your plan?” and offers a binary choice (turn, and continue). During this phase, participants are expected to engage in more individualistic processes by considering what the other might do (mentalizing), evaluating the different possibilities, and planning their behavior accordingly. (b) Dynamic phase represents the car video phase in which participants can decide whether they want to turn (and when to turn within a 5‐s interval) or continue. This phase is expected to be associated with the predicting other's strategy, belief updating, monitoring the other's action, re‐evaluating one's own expectations, and real‐time behavior adjustment. This figure was created with BioRender.com. ACC, anterior cingulate cortex; aIns, anterior insula; Cun, cuneus; Ins, insula; PFC, prefrontal cortex; Prec, precuneus; Str, striatum; TPJ, temporoparietal junction.

At the behavioral level, we expect unilateral cooperation/defection (one‐turning condition) and turn‐taking (Tit‐for‐Tat) strategy to be the most common outcomes (Hernandez‐Pena et al., 2023; Kümmerli et al., 2007; Mantas et al., 2022) and a high choice consistency between the two decision‐making phases (i.e., planning and action). At the neural level, we hypothesize that brain areas previously associated with mentalizing and social decision‐making (i.e., TPJ, precuneus, PFC, insula, or striatum) will be more active during both decision‐making phases compared with baseline (Báez‐Mendoza et al., 2021; Lee & Harris, 2013; Schurz et al., 2014; Van Hoorn et al., 2019). Additionally, as outlined in Figure 1, we assume different cognitive and neural models for each decision‐making phase. Specifically, decision‐making with static context will be more involved in an individualistic process of action planning and evaluation of the different outcomes which will increase activation in areas such as PFC, insula, or striatum (Coutlee & Huettel, 2012; Droutman et al., 2015; Ernst & Paulus, 2005). For dynamic decision‐making (action phase), the information available changes over time which urges participants to update their beliefs and monitor each other's actions becomes more prominent (De Weerd et al., 2022), thus higher activation is expected in mentalizing, belief updating, and monitoring‐related areas (i.e., TPJ/STS, PFC, precuneus or ACC; Assaf et al., 2009; Huber et al., 2015; Kobayashi & Hsu, 2017; Krain et al., 2006; O'Reilly et al., 2013).

Second, we explored differences in brain activation between turning (cooperation/risk avoidance) and continuing (defection) choices during each decision‐making phase. In line with previous game theory literature, cooperation (“turning” decision) is expected to show increased activation in areas such as the insula, PCC, anterior frontal cortex, OFC, superior parietal cortex, and calcarine sulcus compared with defection (“continuing” decision; Decety et al., 2004; Tsoi et al., 2016). In the framework of this task, the decision to turn could also be driven by the motivation to avoid a possible risk (car crash). Deciding on the nonrisky compared with the risky option has been previously associated with PCC/precuneus, bilateral middle temporal gyrus, and bilateral posterior insula (Roy et al., 2011). The opposite contrast (defection > cooperation) is expected to activate more SFG, mPFC, and PCC regions (Fukui et al., 2006). Risky over non‐risky decision is expected to be related to activation in dorsal ACC, superior and middle frontal gyri, striatum, thalamus, precuneus, and anterior insula (Paulus et al., 2003; Roy et al., 2011). We explored cooperation versus defection decision patterns in the different phases, expecting them to be associated with different neural activation patterns depending on the phase (e.g., higher activation in ACC and anterior insula (Krain et al., 2006; Mohr et al., 2010; Morriss et al., 2019; Wu et al., 2021) in defection (continuing), potentially due to greater uncertainty, compared with cooperation (turning) in the dynamic phase).

Third, we explored whether sex, age, or relationship quality of siblings are related to their decision‐making. As preregistered, we hypothesize that competitive behavior will be associated with higher sibling competition relationship (Hernandez‐Pena et al., 2023). Sisters will report better and closer sibling relationship, as well as behave more cooperatively in the task compared with brothers (Hernandez‐Pena et al., 2023; Jensen et al., 2023). Based on previous literature, we also expect sex (brothers) and sibling competition will emerge as positive predictors of brain activity in mentalizing‐related areas during decision‐making (i.e., bothers are expected to show higher activation in mentalizing‐related brain regions; Gao et al., 2019; Krach et al., 2009; Rogers et al., 2018; Veroude et al., 2016).

## 2 METHODS

### 2.1 Participants

Participants were recruited through flyers and online advertisements on social media platforms. Potential participants underwent a screening process to exclude individuals with magnetic resonance imaging (MRI) incompatibilities (e.g., pregnancy or body metals), pharmacological treatment, alcohol or drug abuse, or lifetime history of neurological or psychiatric illness using a short version of the Structured Clinical Interview for DSM‐IV (SCID‐IV) for Axis I disorders (Wittchen & Zaudig, 1997). Inclusion criteria encompassed same‐sex siblings aged between 18 and 35, with a maximum age difference of 5 years, and a minimum of 10 years of co‐residence.

In total, 100 participants (50 same‐sex sibling pairs) were included after screening. Five participants were excluded from analyses after data inspection: two due to excessive head movement (movement above 3 mm was used to exclude participants) during the task and three because of poor performance on the task (more than 80% missing responses to the static decision‐making question). The final sample consisted of 95 participants (49 men, mean age = 22.06 years (SD = 2.81), see Table S1 for further demographic details). In the pairwise analysis, we included 46 complete pairs. Among the five excluded participants, three and their respective partners were removed, and two others formed a single pair, resulting in the exclusion of four pairs.

### 2.2 Procedure

Data collection took place at the RWTH University Hospital Aachen (Germany). Ethical approval was obtained from the Ethics Committee of the University Hospital RWTH Aachen (EK 407/20). All participants provided written consent in accordance with the Declaration of Helsinki prior to participation. Upon arrival, participants received instructions via a prerecorded video to ensure consistency in the explanations. Any uncertainties were subsequently clarified by the experimenter. Participants were asked not to discuss any game strategy with their partner to elicit natural sibling interaction and interpretation of information across events as they occur during the game (Segal & Hershberger, 1999). Participants were informed that they could earn up to 10€ extra in total based on their performance in the last two tasks, based on which of the two siblings achieved the most points. Thereby, we aimed to enhance their motivation to win the game against their opponent and strengthen the competitive context. For ethical reasons due to manipulations by the experimenters in the last task of the study, all participants received the maximum compensation of 60€ after debriefing.

**FIGURE 2:**
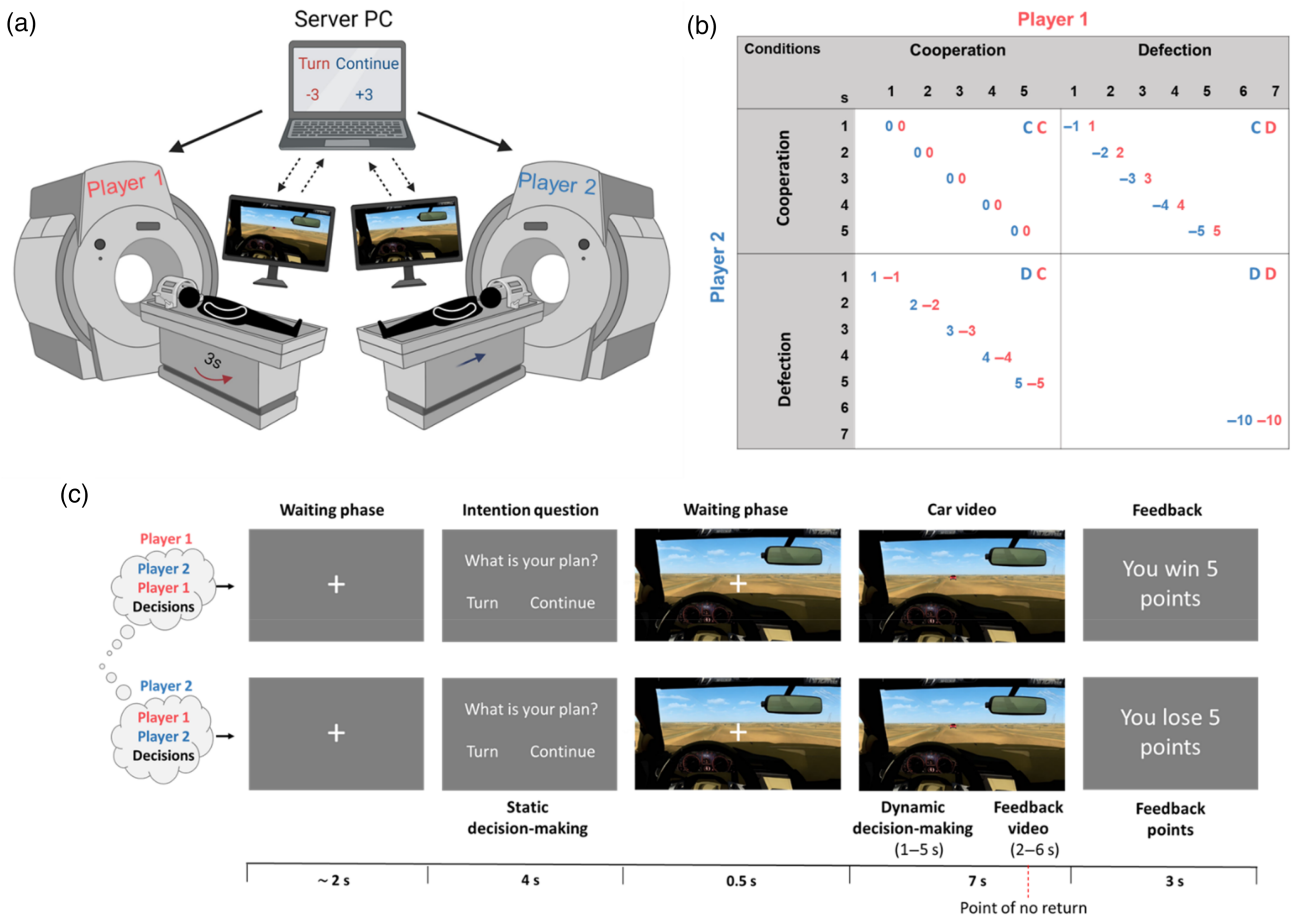
Representation of the hyperscanning setup and the Interactive Chicken Game task. (a) Hyperscanning set‐up. Each scanner was connected to the server PC, which handled the communication to start the scanners simultaneously and managed the trial information from and to the task PCs. This figure was created with BioRender.com. (b) Interactive Chicken Game task payoff matrix. The numbers at the top and left side outside the table represent the seconds across the trial. The numbers inside the table represent the points each participant won or lost, and in the one‐turning condition depending on the waiting time to turn. For one participant, the outcomes are summarized in the following order from better to worse: One‐turning unilateral defection (participant defects/continuous, outcome: 1–5 points); (d) the other cooperates/turns; (c) >mutual cooperation (both turn, outcome: 0 points) (CC) > one‐turning unilateral cooperation (participant turns, outcome: −1 to −5 points); (c) the other continues; (d) >mutual defection (both continue, outcome −10 points) (DD). (c) Trial design. The waiting phase corresponds to a jittered fixation cross, followed by the intention question (static decision‐making process). Before the car video, a fixed brief fixation cross was presented to signal the participants to get ready. The car video included the dynamic decision‐making process and the feedback video with different endings depending on the outcome. Finally, participants received their outcomes in the feedback phase.

Following instructions, each participant was randomly assigned to one of the two scanners (see Figure 2a) and performed three tasks in the same order: the Cooperation Tetris Task, the Interactive Chicken Game task, and the Interactive Taylor Aggression Paradigm. This article focuses exclusively on the Interactive Chicken Game task (for a detailed description of the other tasks, see Hernandez‐Pena et al., 2023; Koch et al., 2024. The whole project aims to explore behavioral and neural differences in sibling pairs across various social interaction tasks. This article focuses solely on social decision‐making, excluding results from the other tasks due to their different goals (forced cooperation and aggression) and designs. Following imaging data collection, participants were guided to two separate rooms to complete self‐report questionnaires using the online tool Sosci Survey (https://www.soscisurvey.de/). The entire procedure lasted ~3 h.

### 2.3 Task

The Interactive Chicken Game task is a validated tool for studying real‐time social interaction in siblings (Hernandez‐Pena et al., 2023). The CG simulates a risky situation where two participants drive cars toward each other; the one who swerves first loses and is labeled as the “chicken.” In the theoretical and traditional version of the CG, participants choose between two options (“cooperate” or “defect,” or similar versions e.g., “C” or “D”) and their gains or losses depend on both choices. From one person's perspective, unilateral defection (i.e., participant chooses to continue/“defect” and the other to turn/“cooperate”) results in the best outcome, followed by mutual cooperation (i.e., both participants choose to turn/“cooperate”), unilateral cooperation (i.e., participant chooses to turn/“cooperate” and the other to continue/“defect”) and mutual defection (both participants choose to continue/“defect”). This paradigm is a valid tool for studying reciprocal mind reading in tense interpersonal interactions (Fukui et al., 2006), and offers higher ecological validity for investigating cooperative versus competitive behavior compared with the Prisoners Dilemma (Butler et al., 2011; Kümmerli et al., 2007; Su et al., 2018). In this context, cooperation is not defined as other economic tasks where there is a direct gain from working together on each trial. By cooperating (i.e., both turning in the same interval of the trial), participants avoid a punishment (car crash), rather than gaining a reward. Also, the iterative aspect of the task makes it possible for cooperation over time to develop through the turn‐taking strategy. In other words, the task is primarily embedded in a competition context, in which cooperation is circumscribed to avoiding a worse outcome and coordinating with the other participant over time in order for both to gain points.

The Interactive Chicken Game task integrates decision‐making in static and dynamic context, and feedback phases (see Figure 2c). During strategic decision‐making in the static phase, participants had to answer the question “what is your plan?” by choosing between turning or continuing. To do so, they conveyed their decisions by pressing either the index or ring finger buttons, respectively, on an MRI‐compatible response device. Initial instructions informed that in this phase participants should report their behavioral intention but could change their decision if they wished in the subsequent driving phase. This phase lasted for 4 s. After a brief fixation cross (0.5 s), the dynamic decision‐making phase began with a car video (7 s). During this phase, participants adopted a first‐person driver perspective on the video of their car driving toward the other participant's car, including the sound of an accelerating engine. They had to decide whether they wanted to turn and when. The decision period was divided into five 1‐s intervals to allow small windows that reflected a turn at the same interval. After 5 s, a “point of no return” was reached where participants could no longer press, and the car crash was imminent; simulating real‐life scenarios where the risk of crashing escalates as the trial progresses. This phase not only increased immersion but also facilitated the exploration of decision‐making within a more interactive framework. This was achieved by having different possible outcomes based on both the participant's decisions and the timing of the decision. This design permitted the investigation of different degrees of cooperation beyond the binary “cooperate” or “defect” option in the static phase. There was no verbal communication between participants in any of the phases.

During the car video phase (see Figure 2c), participants were exposed to different “feedback videos” depending on their decisions. If the participant (or both) chose to turn, the screen displayed the video of the participant's car turning at different intervals (1–5 s). If the other participant opted to turn, the current participant saw the other car turning at different intervals (1–5 s). If neither participant turned, a car crash was shown (2 s). The game structure created the following outcomes: both turning (mutual cooperation), one turning (unilateral cooperation/defection), and both crashing (mutual defection). At the end of each trial, participants received points based on both participants' decisions during the dynamic decision‐making phase (see Figure 2b for the different possibilities and figure caption for a detailed description of outcome points depending on both decisions). The task aim was to earn as many points as possible compared with the sibling. Points were accumulated over time based on “wins” (positive points) and “losses” (negative points), which were then added to or subtracted from the overall total score. Although most of the total game scores were negative, the comparison between siblings relied on choosing the one with the fewest negative points. This instruction offered participants different possible strategies for gaining points; noting that defection is only advantageous if the other turns, but if the other also defects this leads to the worst outcome for both. Given the dyadic structure and iterative approach (multiple rounds rather than a one‐shot game), participants may consider in their current decisions both their own and other's previous choices. Thus, one of the most favorable strategies to maximize both participants' points over time is to use the strategy of turn‐taking. This has been defined as the degree to which an individual's behavior was influenced by the motivation to reciprocate the other participant's level of cooperation in the previous round by calculating the turn‐taking frequency, also known as Tit‐for‐Tat strategy (Pisauro et al., 2022). For this score, we calculated the number of trials in which participants made the same decision as the other participant in the previous trial (not counting both turning or both crashing trials). Dominance CG score represented the sum of the points received by the participants (recoding crashes as +10 points). This score serves as an individual value (compared with the pair outcome scores) using the continuum choices for an estimation of how dominant and competitive participants were during the task (Hernandez‐Pena et al., 2023). Higher scores indicate more competitive behavior.

The paradigm consisted of 45 trials (including three practice trials), divided into three blocks (total task duration between 16 and 20 min, variance depending on the duration of the questions phase). After each block and at the end of the task, participants answered questions related to their experience and motivations during the game (see Table S2). During the brief interview at the end of the study, none of the participants questioned the authenticity of playing with their siblings. The paradigm was programmed using PsychoPy3 (version 2020.2.10, Peirce et al., 2019; https://www.psychopy.org/).

### 2.4 Imaging data acquisition

Data were collected using two Siemens 3‐Tesla MRI scanners equipped with 20‐channel head coils (one Siemens MAGNETOM Prisma scanner and one MAGNETOM Prisma fit scanner, Siemens Medical Systems, Germany). The MRI scanners were technically aligned in terms of field strength, gradients, and software. The scanners, located in the same hospital, were interconnected through a virtual server hosted at the Brain Imaging Facility of the University Hospital RWTH Aachen. Communication between tasks was managed by the server using the Socket module (Python 3.6.6) via TCP/IP. To synchronize the scanners and task performance, the computers that displayed the paradigm (hereafter referred to as “clients”) at each scanner were continuously communicating with the server (see Figure 2a). The server simultaneously dispatched a message to both clients to trigger the respective scanner through a parallel port. This ensured that both imaging data and task‐related data acquisitions were synchronized with a maximum latency of ~16 ms. At the start of the task and each trial, the server waited for confirmation from both clients to verify task synchronization. Upon receiving specific messages from both clients (e.g., trial‐related information), the server responded to both clients with appropriate messages.

Functional images were acquired using a T2*‐weighted echo‐planar imaging (EPI) multiband sequence with the following acquisition parameters: repetition time (TR) = 1500 ms, time to echo (TE) = 34 ms, multiband acceleration factor = 3, flip angle = 70°, field of view (FOV) = 192 × 192 mm^2^, matrix size = 96 × 96, 69 slices, interleaved, voxel size = 2 mm^3^. Slices covered the whole brain and were positioned transaxially parallel to the anterior–posterior commissural line and acquired posterior to anterior. Task and functional scans lasted ~16–20 min (total study scan length ranged approximately between 51 and 62 min). Structural images were acquired using a T1‐weighted MPRAGE sequence with the following acquisition parameters: TR = 2000 ms, TE = 3.03 ms, flip angle = 9°, FOV = 256 × 256 mm^2^, voxel size = 1 mm^3^, interleaved, distance factor: 50%. The gradient‐echo sequence to generate the field map had the next characteristics: TR = 526 ms, short TE = 4.87 ms, long TE = 7.33 ms, flip angle = 60°, voxel size = 3 mm^3^, FOV = 192 × 192 mm^2^.

### 2.5 Questionnaires

To explore the sibling relationship, participants completed the Sibling Type Questionnaire (STQ, Stewart et al., 2001). To assess aggressive, competitive, dominance and impulsivity traits, participants completed the Buss‐Perry Aggression Questionnaire (Buss & Perry, 1992), Hypercompetitive Attitudes Scale (Ryckman et al., 1990), Personal Development Competitive Attitudes Scale (Ryckman et al., 1996), Dominance, Prestige and Leadership Motives Scale (Suessenbach et al., 2019), Rank Style with Peers Questionnaire (Zuroff et al., 2010), Sense of Power Scale (Anderson et al., 2012), Machiavellianism Scale (Mach IV; Christie & Geis, 1970), and Barratt Impulsiveness Scale (Patton et al., 1995).

### 2.6 Behavioral analysis

All analyses were performed using Matlab (v2017b, MathWorks, Natick, MA, USA), SPSS (IBM SPSS Statistics 20.0, Chicago, IL, USA), and GraphPad Prism 9.5.1 (only the block‐wise time effect analyses; GraphPad Software, Boston, MA, USA, https://www.graphpad.com/). Shapiro–Wilk and Levene's tests statistics were applied to check for the assumption of normality and homogeneity of variances, respectively. Nonparametric tests were used when the assumption of normality was violated. For all analyses, a significance threshold of *α* = .05 (two‐tailed) was used. Post hoc analyses were corrected for multiple comparisons using the Bonferroni method.

Chi‐square tests were used to analyze differences in the frequency of cooperation and defection choices trials both within and between each decision phase, across all trials. Non‐parametric Wilcoxon tests were calculated to examine the differences in frequency between dyads' outcomes (both turning, both crashing, one‐turning, and turn‐taking) within and between the decision phases due to the non‐normal distribution of these variables.

We calculated the proportion of trials in which participants maintained consistency in their decisions (consistent cooperation: cooperate in both static and dynamic decision‐making; consistent defection: defect in both static and dynamic phases) and trials with inconsistency (inconsistent cooperation: cooperate in the static phase and defect in the dynamic phase; inconsistent defection: defect in the static phase and cooperate in the dynamic phase). We ran Wilcoxon tests to discern potential differences between these conditions across all participants.

To investigate potential differences in the distribution of feedback points across all trials during the dynamic decision‐making phase, we applied a Kolmogorov–Smirnov test. Additionally, a Chi‐square test was conducted to analyze the distribution of the decision phase question choices at the end of the task across all participants (see description of the question and choices in Table S2). To explore differences in trial‐by‐trial RTs over time, we performed two linear mixed models. First, we conducted a linear mixed model using RT in the static decision‐making phase as dependent variable, and static decision‐making choice (0 = continue, 1 = turn), dynamic decision‐making choice (0 = continue, 1 = turn), and the interaction as fixed factors, and trial number as covariate. Subject ID was included as random intercept. In the second linear mixed model, we only included RT of the turning trials in the dynamic decision‐making phase, since continuing trials have an RT of 0. Therefore, we did not include the conditions as factors in this model, but only trial number as covariate and subject ID as random intercept. The estimation method was restricted maximum likelihood. The effect of time on dyad's outcome, individual RTs when turning, and participant's feelings across blocks have also been analyzed in a block‐wise approach; see Section S3 for further analysis description. Spearman's correlation was calculated (corrected significant level *α* = .0018) to explore the relationship between behavior and task strategies reported at the end of the whole task (for specific strategic statements consult Table S2).

To explore potential sex differences in behavior (across dyads), and sibling relationship and personality traits (across individuals), we conducted independent sample *t*‐tests and Mann–Whitney *U* tests (for variables that were not normally distributed). Demographic differences were examined using Chi‐square tests across participants. We conducted a linear mixed model using dominance CG score as dependent variable, and sex (1 = man, 2 = woman), sibling competition (competition subscale from STQ) *z*‐scores, and age difference in months as predictors across participants. Pair ID was included as random intercept to account for the nested design. The estimation method was restricted maximum likelihood.

### 2.7 fMRI data analysis

The first three volumes were discarded to mitigate signal changes due to radiofrequency excitation. Functional images were preprocessed and analyzed with the Statistical Parametric Mapping Software (SPM12 software, http://www.fil.ion.ucl.ac.uk/spm/). First, a voxel displacement map was created using the gradient‐echo sequences and the default mask brain image from SPM. The precalculated phase map was applied to the EPI scans for distortion correction in the realignment step to mitigate the inhomogeneities induced by the magnetic field. Unwarped EPI scans were realigned to the first volume as a reference. Each individual anatomical scan was co‐registered to its corresponding mean EPI scan. Using the tissue probability map from SPM, the anatomy scans were normalized to MNI152 standard space. The same normalization parameters were applied to the EPI scans, transforming them into the MNI152 space and resampling them to a voxel size of 2 mm^3^. To improve the signal‐to‐noise ratio, the EPI scans were smoothed using an isotropic Gaussian kernel of 6 mm full‐width‐at‐half‐maximum.

At the individual level, the time‐series from each participant were fitted to a general linear model with a task‐specific model (first level). In total, we defined nine regressors (see Figure 2c): static decision‐making (turn and continue, separately), dynamic decision‐making (turn and continue, separately), feedback video (win and lose, separately), and feedback points (win and lose, separately). Another regressor of no interest was included to encompass missing trials in the static decision‐making question. The duration of each condition was measured from the onset of each condition to the onset of the next phase and modeled as mini‐epochs (for phase duration see Figure 2c). Jitter and intertrial intervals were captured by implicit baseline. The nine stimulus regressors were convolved with the canonical hemodynamic response function (HRF) with no derivatives. A high‐pass filter of 128 s was applied to remove low‐frequency drifts. In addition, the six realignment parameters were added to capture residual movement‐related artifacts.

For group analyses (second level), we used the full factorial design of SPM12. A 2 × 2 design included two factors with two levels each: decision‐making factor (static and dynamic) and choice (turn and continue), including scanner as covariate. Our interest was to analyze the main effect between static decision‐making and dynamic decision‐making and the comparison between turning (cooperation) and continuing (defection) within each phase. A conjunction analysis was performed to identify brain regions demonstrating consistent activation across the two decision‐making phases. Individual contrasts were defined for each condition (static decision‐making vs. baseline, and dynamic decision‐making vs. baseline) and included together for the conjunction analysis. Whole brain analyses were conducted with a threshold of *p* < .05 with a family‐wise error correction (to correct for multiple comparisons) on a voxel level.

Sex differences were explored at the whole brain level with independent‐sample t‐tests for each condition (static decision‐making turn/cooperation, static decision‐making continue/defection, dynamic decision‐making turn/cooperation, and dynamic decision‐making continue/defection), including scanner as covariate. Given the exploratory nature of these analyses, they were conducted at *p* < .05 with family‐wise error (FWE) correction on a voxel level, but also at a cluster‐defining threshold of *p* < .001 with FWE‐corrected *p* < .05 on a cluster level.

We conducted a hypothesis‐driven region of interest (ROI) analysis in core regions of the theory of mind network. For this, we used the uniformity test mask (from now on called “mentalizing mask”) from the automated Neurosynth meta‐analysis under the term “mentalizing” which included 151 neuroimaging studies (see Figure S4 and https://neurosynth.org/analyses/terms/mentalizing/). We applied a threshold of *p* < .05 with family‐wise error correction on a voxel level.

To explore the relationship between any significant activation in the ROI analyses (*k* > 20 voxels) of the mentalizing areas during decision‐making in general (conjunction analysis) and sibling relationship, sex, and age difference, we extracted the average beta values for each particular ROI (from the overlap between the significant brain activation in the conjunction analysis and the mentalizing mask). Individual beta values were extracted per condition (static decision‐making turn, static decision‐making continue, dynamic decision‐making turn, and dynamic decision‐making continue) using a threshold of *p* < .50 (to include voxels that do not show only relevant activation and avoid results obtained by a few participants with very high activation patterns).

Linear mixed models were calculated for each extracted ROI per condition, see above, including the ROI's individual beta value as dependent variable, and sex (1 = man, 2 = woman), sibling competition STQ *z*‐score, and age difference in months as predictors. To account for the nested design of our data, pair ID was included as random intercept. The estimation method was restricted maximum likelihood. We corrected for multiple comparisons between ROIs within the same contrast (corrected *α* = .007).

## 3 RESULTS

### 3.1 Behavioral results

**FIGURE 3:**
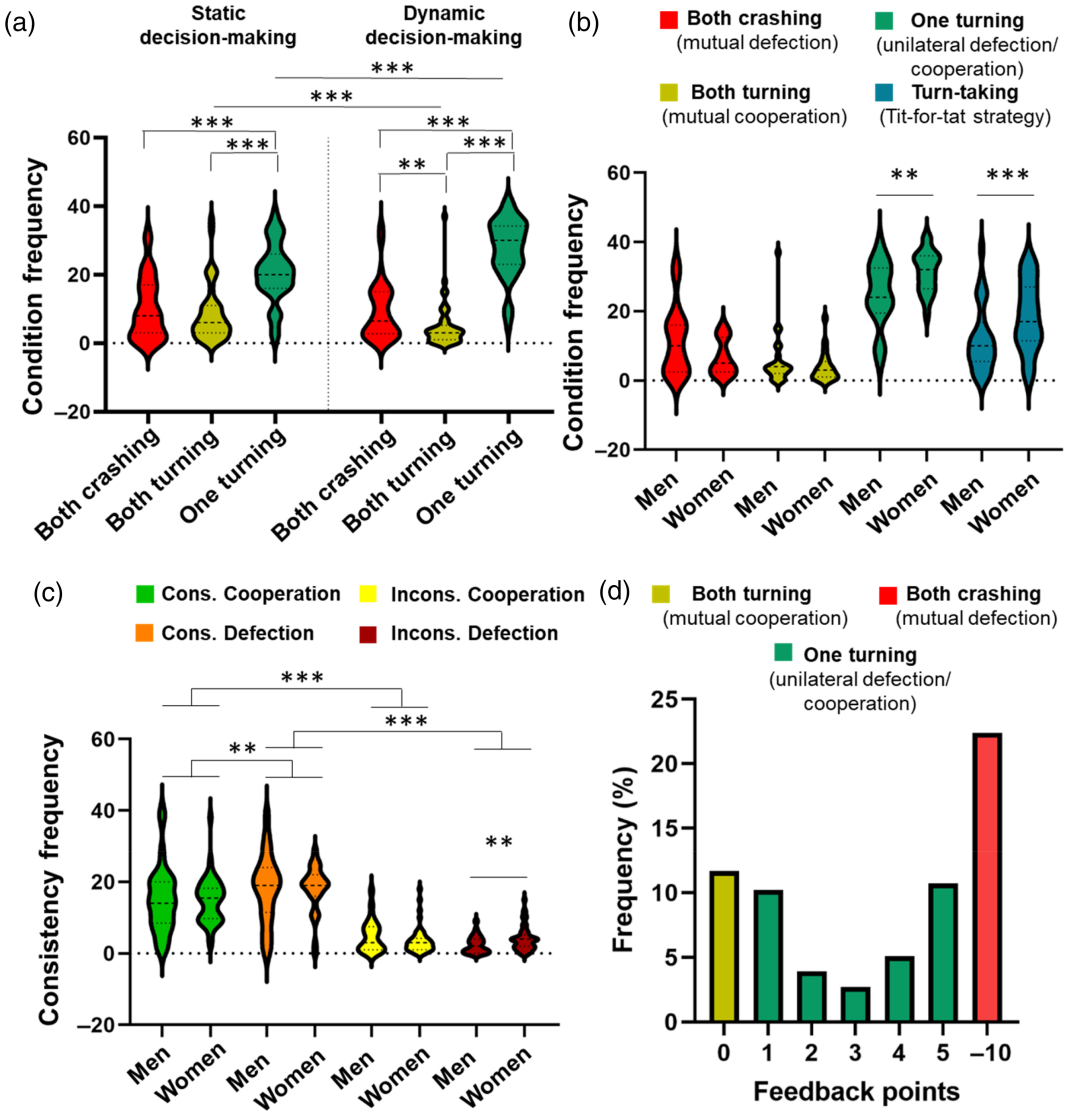
(a) Comparison of conditions (both crashing, both turning, and one turning) between and within decision‐making phases. The violin plots depict summary statistics and the kernel density estimation to show the frequency distribution of each condition. In the middle of each density curve, the thick dotted line represents the median, while the thinner solid lines represent the quartiles. (b) Distribution of outcome conditions based on the decision in the dynamic phase, and sex differences between them. (c) Distribution of consistency in the choices between the static and dynamic decision‐making phases, and sex differences. Cons., consistent; Incons, inconsistent. (d) Frequency of the feedback points across trials depending on the RT and choices from both participants. *Y*‐axis scale: 0 corresponds to the *both turning* condition. 1, 2, 3, 4, and 5 (−1, −2, −3, −4, −5 represent the same frequency mirrored for the other participant, so it was omitted) correspond to the *one‐turning* condition for each second of the turning RTs. −10 corresponds to the *both crashing* condition. *p < .05, **p < .01, ***p < .001.

For comparisons between frequency of turning (cooperation) and continuing (defection) trials and conditions between and within phases and specific statistics, see Section S4. As summary of the main results, continuing (defect) was significantly more chosen than turning (cooperation) in both phases across trials, although the difference was small (see Table S3). The one‐turning condition was the most prevalent one, with significant differences with the other conditions within each phase (see Figure 3a). The phases did not differ in the amount of both crashing trials (*p* > .05), but the static decision‐making phase had a higher frequency of both turning trials, while the dynamic decision‐making phase had a higher frequency of one‐turning trials (see Table S3). Participants indicated that they made their decisions mainly during the static phase. In general, participants were more consistent than inconsistent in their choices (see Figure 3c and Table S4), with higher consistency in continuing (defection) than in turning (cooperation) trials. Specific patterns of behavior were associated with game strategies, particularly, both crashing condition was significantly positively associated with the perception of the sibling being aggressive, and negatively with the perception of fairness. Turn‐taking strategy was significantly positively associated with the perception of fairness and coordination (see Table S5 for statistical details).

There was a significant difference in the distribution of feedback points (*z* = 10.24, *p* < .001), showing a higher frequency at the extremes (see Figure 3d). There was a significant effect of time on RT, in which participants answered faster over time in the static decision‐making question, but also decided to turn earlier in the dynamic decision‐making phase. Also, there was a significant interaction effect in the linear mixed model on RT during static decision‐making, in which incongruent (change of choice) conditions required more time to answer compared with congruent conditions. Similar results were obtained with the block‐wise analysis, in which participants turned later in the dynamic phase in the first block compared with the other two blocks. Pairs decided to crash more frequently and performed unilateral turning less frequently in the first block compared with the second block. In addition, there was a decrease in feelings of guilt and empowerment over the task. For the specific results and discussion of the effect of time in the task, consult Sections S5 and S6, and Tables S6–S8 as well as, Figures S2 and S3.

#### 3.1.1 Sex differences

No sex differences were observed for the demographic variable (see Table S1 for statistics). Sisters had significantly higher scores for mutuality and longing compared with brothers, whereas brothers had higher apathy scores. Across pairs, men scored higher than women for dominance traits and leadership style, Machiavellianism, and impulsivity (see Table S9 for statistics).

Sex differences were evident in behavior during the Interactive Chicken Game task (see Table S4 for statistics). Sister pairs demonstrated higher rates of unilateral cooperation/defection and turn‐taking strategy (see Figure 3b). Consequently, men had significantly higher dominance CG scores. There were no significant differences in mutual cooperation and mutual defection. Women exhibited significantly more inconsistency between the two phases after planning to defect than men (see Figure 3c). There was no significant difference in RT when turning.

The results of the linear mixed model analysis on competitive behavior revealed that sex significantly predicted dominance CG scores (*F*[1, 43.02] = 4.68, *p* = .036), with men showing higher scores. Neither sibling competition relationship (competition subscale from STQ) scores (*F*[1, 71.22] = 1.50, *p* = .225), nor age difference (*F*[1, 43.18] = .33, *p* = .557) were significant predictors of dominance CG score. For further details on the linear mixed model results (see Table S10).

### 3.2 fMRI results

In the following sections, individual brain activation results are presented. No interbrain analyses have been performed and included in this article.

#### 3.2.1 Whole‐brain analysis

**FIGURE 4:**
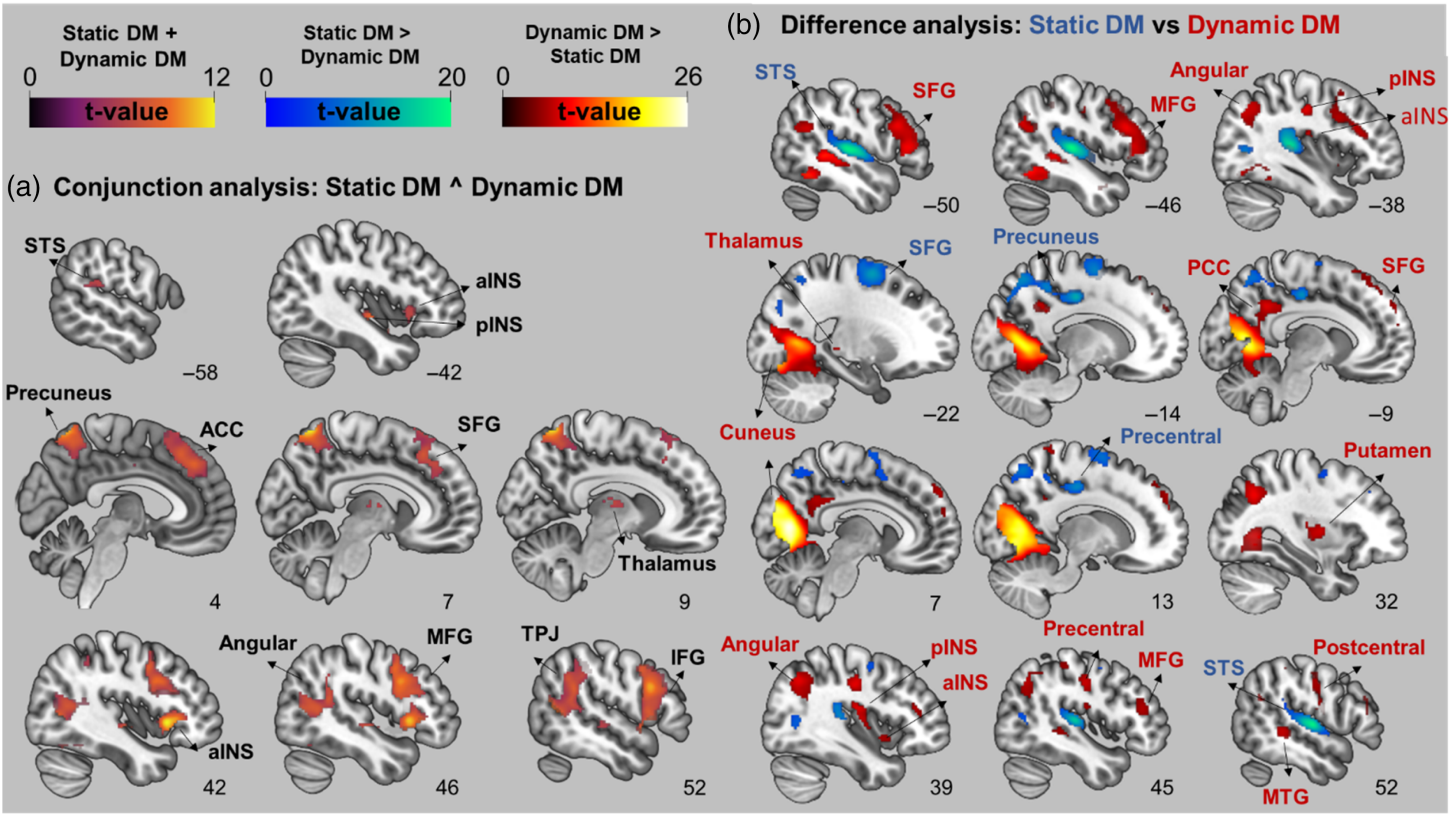
(a) Conjunction analysis results showing the shared activation between static decision‐making and dynamic decision‐making. (b) T‐contrasts of static decision‐making versus dynamic decision‐making. The statistical threshold map is set to *p* < .05, family‐wise error‐corrected at voxel level. The figure was created with MRIcroGL1.2. software, and the coordinate values (sagittal plane) corresponding to the standard 2D slice coordinate system of the software. ACC, anterior cingulate cortex; aINS, anterior insula; DM, decision‐making; IFG, inferior frontal gyrus; MFG, middle frontal gyrus; MTG, middle temporal gyrus; PCC, posterior cingulate cortex; pINS, posterior insula; SFG, superior frontal gyrus; STS, superior temporal sulcus; TPJ, temporoparietal junction.

On the whole brain level of the conjunction analysis (static decision‐making ∩ dynamic decision‐making), significant shared activity emerged in the right TPJ, left OFC, left insula, right thalamus, and ACC (see Figure 4a and Table S11). As shown in Figure 4b and Table S11, static decision‐making > dynamic decision‐making revealed increased activity in bilateral superior temporal gyri, superior frontal gyri, putamen and caudate, among others. For the reverse contrast (i.e., dynamic decision‐making > static decision‐making), increased activity was found in the bilateral cuneus, middle temporal gyri, amygdala, frontal poles, ACC, left TPJ and left inferior frontal gyrus (IFG).

**FIGURE 5:**
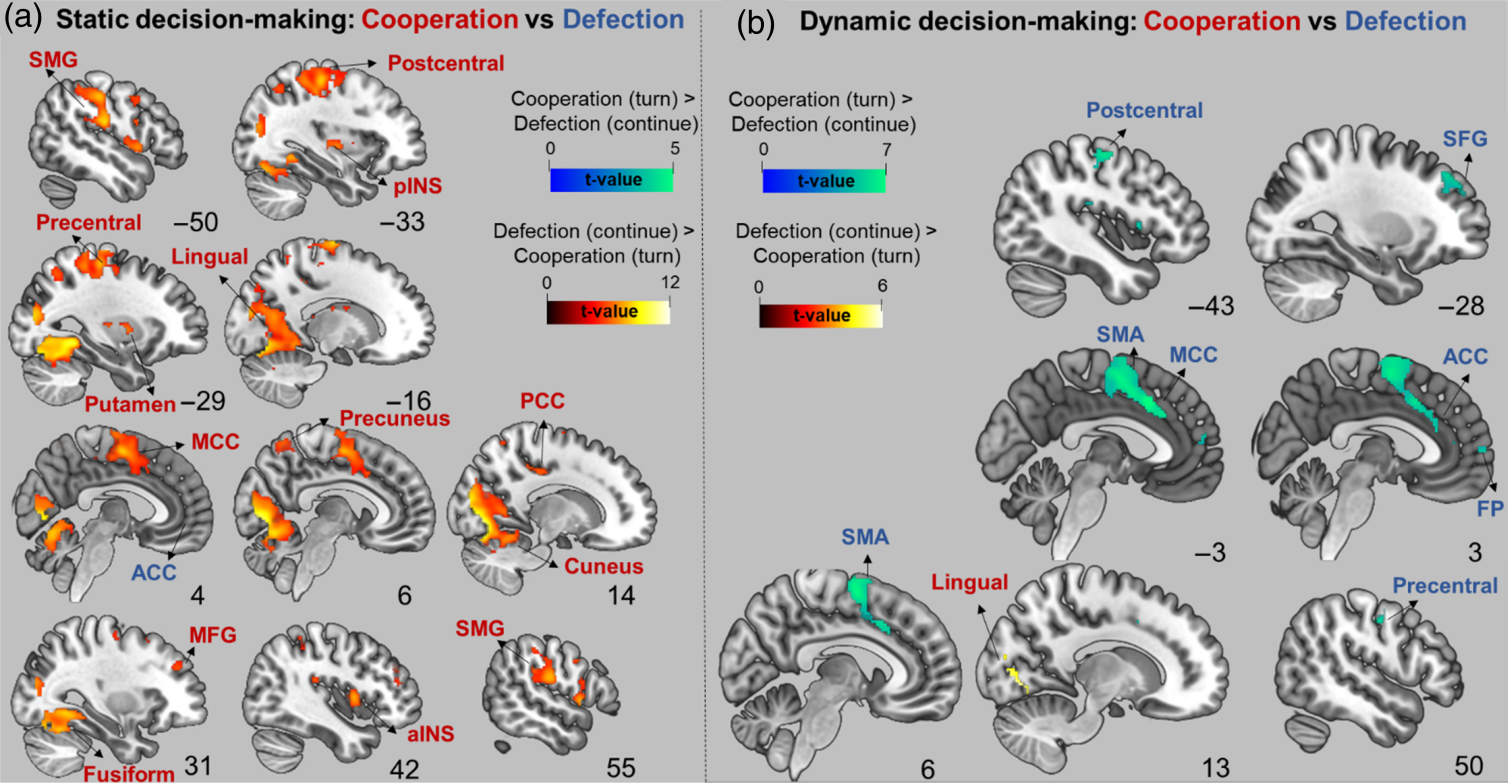
(a) Static decision‐making turning (cooperation) versus continuing (defection) contrast. (b) Dynamic decision‐making turning (cooperation) versus continuing (defection) contrast. The statistical threshold map is set to *p* < .05, family‐wise error corrected at voxel level. The figure was created with MRIcroGL1.2. software, and the coordinate values (sagittal plane) corresponding to the standard 2D slice coordinate system of the software. ACC, anterior cingulate cortex; aINS, anterior insula; DM, decision‐making; FP, frontal pole; MCC, midcingulate cortex; MFG, middle frontal gyrus; PCC, posterior cingulate cortex; pINS, posterior insula; SMA, supplementary motor area; SMG, supramarginal gyrus.

In the static decision‐making phase, turning (cooperation) > continuing (defection) exhibited significant activation in various brain areas, including the bilateral fusiform, precentral and postcentral gyri, superior frontal gyri, right supramarginal gyrus (SMG), left caudate, putamen, and thalamus. The reversed contrast demonstrated significant activation in a small cluster within ACC (see Figure 5a and Table S12).

During dynamic decision‐making, turning (cooperation) induced higher activation in the right lingual gyrus and frontal pole compared with continuing (defection). For continuing (defection) > turning (cooperation), increased activity was observed in the bilateral paracingulate gyri, ACC, precentral gyri, frontal pole, superior frontal gyri, right putamen, and amygdala, among others (see Figure 5b and Table S12).

#### 3.2.2 Region of interest analysis

A conjunction analysis using the mentalizing mask (see Section 2) revealed significant activation in several regions, including bilateral insula and the right TPJ, IFG, SFG, and thalamus (see Figure S4 and Table S13). In the static decision‐making > dynamic decision‐making contrast, significant activation was limited to four small clusters located in the bilateral lateral occipital cortices, left precentral and right paracingulate gyri. The reversed contrast (dynamic decision‐making > static decision‐making) showed increased activation in numerous regions, including the bilateral precuneus, PCC, amygdala, middle temporal gyri, frontal poles, and left IFG (for further details, see Table S13). Dynamic decision‐making engaged more activation in mentalizing‐related regions confirmed by the higher overlap with the mentalizing mask (see Figure S4).

#### 3.2.3 Sex differences results

Exploratory whole brain analysis revealed higher activation in men compared with women in the right SFG during static decision‐making (turning > continuing), bilateral fusiform gyri, SMG, right hippocampus and left thalamus (static decision‐making continuing > turning), as well as bilateral precuneus gyri and left SMG (dynamic decision‐making continuing > turning), among others (see Figure S5 and Table S14).

#### 3.2.4 Linear mixed model results

After correcting for multiple comparisons (corrected *α* = .007) per contrast, only sex (men) remained a significantly positive predictor for activity in the right TPJ during dynamic decision‐making in both turning (cooperation) and continuing (defection) conditions. Uncorrected significant results of linear mixed model on ROIs (right IFG, right and left insula, right TPJ, right middle frontal gyrus, right precuneus, and right SFG) are described below; for nonsignificant results, please refer to Table S10. Sex (men) was a significant positive predictor of activity in the right TPJ (*F*[1, 40.60] = 8.04, *p* = .007), middle frontal gyrus (*F*[1, 42.93] = 5.52, *p* = .023), and SFG (*F*[1, 42.69] = 5.49, *p* = .024) during dynamic decision‐making turning, and the right insula (*F*[1, 40.85] = 4.46, *p* = .041), and TPJ (*F*[1, 41.68] = 8.59, *p* = .005) during dynamic decision‐making continuing. Sibling competition relationship (competition subscale from STQ) *z*‐scores significantly positively predicted activation in the right IFG (*F*[1, 87.63] = 5.48, *p* = .022), middle frontal gyrus (*F*[1, 87.72] = 4.30, *p* = .041), and precuneus (*F*[1, 88.00] = 4.79, *p* = .031) during dynamic decision‐making turning. Age difference was a significant positive predictor of activation in the right middle frontal gyrus (*F*[1, 43.06] = 4.30, *p* = .044) during static decision‐making turning.

## 4 DISCUSSION

We aimed to elucidate the neural underpinnings of social decision‐making in a competitive context and to examine their association with sibling relationships. During decision‐making, we compared different phases (static = nonchanging prior information; dynamic = changing information requiring belief updating). During the Interactive Chicken Game task, we found similarities in both static and dynamic decision‐making phases, with activation in areas previously linked to mentalizing (predicting other's intentions). Differences between phases were characterized by higher activation in areas related to action planning and outcome evaluation during the static phase, while dynamic decision‐making required more activation in areas often associated with mentalizing (stronger activation compared with the static phase), monitoring of other's behavior, expectation reevaluation, action adaptation, and emotion and visual processing. Cooperation (turn), compared with defection (continue), during static decision‐making involved activation in regions related to mentalizing, while defection (continue), related to cooperation (turn), in the dynamic phase significantly activated areas usually associated with conflict monitoring. Sex differences were found both at behavioral and neural levels, with men behaving less cooperatively and showing higher activation in the TPJ, precuneus, and hippocampus areas, among others. Competitive sibling relationship did not predict competitive behavior in the task but showed a tendency to predict brain activation in frontal and precuneus regions during dynamic decision‐making (not surviving correction for multiple comparisons).

### 4.1 Behavioral findings

As anticipated, participants maintained their planned decisions across trials, consistent with self‐reported strategies. That is, the majority of participants reported using the static decision phase to form decisions. However, participants sometimes deviated from their initial plans during the dynamic phase. This finding may be attributed to the real time and interactive nature of the situation, heightened anxiety about undesirable outcomes, lack of confidence (Pescetelli et al., 2021), or regret over their initial choice (Kirkebøen et al., 2013). Speculatively, participants who initially planned to defect and later decided to turn during the dynamic phase might have done so because their expectation that their partner would turn was unmet. In this case, a reconsideration prevented a car crash (i.e., the worst outcome). Conversely, participants who planned to turn but we observed that they continued driving may have failed to synchronize with their partner to turn at the same interval (i.e., the other turned faster), or changed their mind. Our findings highlight the importance of distinguishing between intentions and real‐time responses in interactive settings. Unfulfilled initial expectations (e.g., the opponent will turn) may require reevaluation and adaptation.

Consistent with prior studies (Kümmerli et al., 2007; Mantas et al., 2022), unilateral cooperation/defection and turn‐taking (Tit‐for‐Tat) strategies were the most prevalent among sibling dyads in our study. Mutual cooperation occurred less frequently during the dynamic phase compared with the static phase which is likely a result of failed synchronization between participants of the decision to “turn” within the same interval. Continue (defect) was slightly more chosen than turn (cooperation/risk avoidance) across trials in both phases, which underlines the competitive context. We observed a tendency to “turn” at either the beginning or end of the trial (before the point of no return), which may reflect a proactive signal communicating cooperative intentions. Conversely, the late turn decision likely relates to optimizing the maximum dyad's outcome by letting the other earn the maximum possible amount of points. However, the occurrence of intermediate turns highlights the notion that cooperation and competition form a continuous spectrum, rather than a strict dichotomy (Pisauro et al., 2022). We observed a change in behavioral strategies, in which participants exhibited more cooperative behavior over time (see Section S6 for further discussion). In addition, participants showed a tendency to turn later in the first block compared with the other blocks, which could point in the direction of a learning effect or change of strategy.

### 4.2 Neural correlates of decision‐making in static and dynamic contexts

Social interactions in a competitive context require implicit “online” mentalization (Assaf et al., 2009). Competition implies more strongly mentalizing skills, compared with cooperation, when social partners have divergent goals (Decety et al., 2004). Therefore, we expected a crucial role of the mentalizing brain network during our task. Decision‐making in static and dynamic environments had shared activation in expected areas related to mentalizing or social decision‐making, including the TPJ, precuneus, posterior STS, mPFC, ACC, insula, OFC, and thalamus (Báez‐Mendoza et al., 2021; Lee & Harris, 2013; Schurz et al., 2014). Both phases required participants to consider the intentions of the other participant, make predictions (mentalizing), and decide between different outcomes. Taking the mentalizing model described by Frith and Frith (2023), the posterior STS together with the TPJ would be functioning as entry hub of the mentalizing system and serve a crucial role in prediction and monitoring one's own and other's actions. The precuneus and PCC in this model act as the “navigator” in the social context and the mPFC and ACC are the “controller” which are essential for accounting for prior expectations, with a relevant role in human–human interactions. In the decision‐making process, the evaluation of potential outcomes is usually associated with activation in anterior insula (Droutman et al., 2015), thalamus (Luo, 2018), and OFC (Khani & Rainer, 2016; Morelli et al., 2022; Serra, 2021). Behavioral action selection involves often the thalamus (Kühn & Brass, 2009) and insula (Droutman et al., 2015).

As expected, decision‐making during static context showed increased activation, compared with dynamic decision‐making, in frontal regions such as superior and middle frontal gyri, precentral gyrus, but also in STS, and striatum. Decision‐making in the static phase (no change in the information available) was assumed to be more involved in an individualistic process of behavior planning thus expecting increased activity of regions supporting action planning. Indeed, superior frontal and precentral gyri are usually involved in action planning using the information from the frontal motor areas to guide behavior (Andersen & Cui, 2009). These areas were also associated with anticipation before feedback following defection compared with cooperation decisions in a PD task (Thompson et al., 2021). The processing of possible outcomes of the decision activates the mesolimbic reward system (Declerck et al., 2013), including the caudate nucleus and putamen, as observed in the current study. Previous literature highlights the involvement of these areas in outcome evaluation, especially in processing of valence and magnitude of rewards (Luo, 2018; Morelli et al., 2022). The putamen is also implicated in representing the action value helping with action decision (Khani & Rainer, 2016). Interestingly, the putamen is more activated when making prosocial decisions with friends compared with disliked peers (Schreuders et al., 2018). We found that participants took longer to make a decision in this phase in incongruent trials (i.e., in which later they change their decision), which might be interpreted as a lack of confidence in their decisions. This could be associated with increased activation in mentalizing‐related regions, as uncertainty about the other's intentions could be greater (Rusch et al., 2020), and greater demands on executive functions including self‐regulation (Hamilton et al., 2011). However, in our data, there are not enough incongruent events per participant to further explore this promising line of research.

In contrast to decision‐making in the static context, dynamic decision‐making, as hypothesized, showed stronger activation in mentalizing‐related regions including the left TPJ, bilateral SMG, and PFC (Molenberghs et al., 2016; Schurz et al., 2014; Van Overwalle, 2009). This was also confirmed by the higher overlap with the mentalizing mask. This could be attributed to several reasons. First, participants were making the final decisions with direct consequences on the trial outcome, which might increase the need to accurately predict other's motivational states. Second, in this dynamic environment, there is continuous feedback about the other's action, therefore, tracking the other's intentions, continuously updating one's beliefs and expectations, and optimally adapting one's own actions become more relevant (Rusch et al., 2020). This probably also involves the activation of ACC, as we observed, due to its role in evaluating and predicting other's behaviors and motivations, monitoring conflicts and errors, and adjusting behavior in response to partner's actions (Apps et al., 2016; Carter & Van Veen, 2007; Pisauro et al., 2022), STS, precuneus, SFG and MFG related to belief updating (Huber et al., 2015; Kobayashi & Hsu, 2017) and dorsal mPFC involved in performance adjustments (Ridderinkhof et al., 2004). Third, this phase incorporated a richer game structure, which could increase participants' engagement and immersion within the decision‐making and mentalizing process (Byom & Mutlu, 2013; Rusch et al., 2020). The higher relevance of visual input in this phase probably involved the strong activation we saw in occipital areas including cuneus and lingual gyrus. Furthermore, this virtual environment with new updating information and, potentially, active engagement could also be translated into increased value and emotion processing, which might implicate the observed activation in the insula, amygdala, and putamen (Ruff & Fehr, 2014). Anterior insula is also associated with choice and timing of actions, related to the decision to act as well as action inhibition (Droutman et al., 2015; Kühn & Brass, 2009), which is more relevant for dynamic, compared with static decision‐making.

Nevertheless, it is important to highlight that differences between the phases might be related to other factors such as more time pressure, cognitive load, or a more vivid setting. Previous studies have observed that scene imagery promoted cooperative behavior, interacting with theory of mind (Gaesser et al., 2018).

### 4.3 Neural correlates of turning (cooperation) versus continuing (defection) within each decision‐making phase

A continuous setting is critical for identifying the neural correlates underlying cooperative and competitive behavior (Pisauro et al., 2022). Expanding previous knowledge on social decision‐making, we found different brain activation patterns of cooperation (turning) and defection (continuing) depending on the decision phase. Neural findings from the static decision‐making phase are similar to those described in previous game theory tasks. As prior literature, cooperation compared with defection, activated superior parietal cortex, PCC, insula, frontal pole, and calcarine sulcus (Decety et al., 2004; Thompson et al., 2021). Cooperation also significantly involved more mentalizing‐related regions compared with defection such as precuneus, SMG, and reward‐related regions such as caudate nucleus and putamen. Comparable activation in mentalizing regions (TPJ and precuneus) was also identified in cooperation over competition (Tsoi et al., 2016), but these results should be interpreted with caution, as they are derived from uncorrected ROI analyses. According to our task design, cooperation is defined as prosocial behavior in which one person possibly allows the other to get a reward at the cost of a likely losing outcome, and perhaps expectation of reciprocal cooperation in the subsequent interactions. Prosocial actions require self‐regulation to inhibit selfish impulses, the mentalizing ability to refocus one's attention to the needs and aims of others, and possibly even a sense of reward (Schreuders et al., 2018). Thus, this empathic, “thinking‐for‐the‐other” and perhaps reciprocal decision might underlie the increased activation in mentalizing and reward‐related regions we found. Another possible interpretation is that activation in the PCC, precuneus, and posterior insula during turning, as opposed to “continuing driving,” is associated with risk avoidance processing as pointed out in other studies (Roy et al., 2011). In contrast, defection over cooperation only activated the right dorsal ACC. The cingulate cortex was also uniquely reported for defection during a theoretical version of the CG, but in a more ventral location (Fukui et al., 2006), interpreted as having a particular role in risky decision‐making and error monitoring.

During dynamic decision‐making, opting for cooperation over defection was associated with notably reduced neural activation relative to the static phase. One plausible interpretation, congruent with the observed behavioral patterns, suggests that individuals who had the intention to cooperate, took their decision in the static phase, requiring less reevaluation, and primarily focusing in the dynamic phase on the timing of the turn. This could explain the observed activation in the cuneus and frontal pole that may be associated with increased significance of visual input in determining the cars' positions and deciding when to turn. On the contrary, deciding to defect, compared with cooperate, significantly activated more brain regions. Based on the task design, and as found in prior literature (de Heus et al., 2010), defecting is the riskier option (leading to either winning, if the other turns, or losing the maximum amount). Therefore, participants who decide to continue might think more strongly about other's intentions (mentalizing) over time. This interpretation should be considered with caution because enhanced mentalizing skills might be required by participants to update their beliefs and change their decision (from continue to turn).

In this phase, and especially during the decision to continue possibly because of being riskier (de Heus et al., 2010), monitoring others' actions and constant reevaluation of own's expectations may become more relevant. A huge cluster including ACC and MCC was found during the decision to defect (continue) over cooperation. As described before, this area is involved in conflict monitoring, especially when there are competing motives (Declerck et al., 2013). A wide range of previous literature associates ACC activity with uncertainty evaluation (Catena et al., 2012; Jiang et al., 2022; Krain et al., 2006; Stern et al., 2010). Activation of the ACC, SFG, and striatum has also been associated with choosing risky over nonrisky options (Roy et al., 2011). Higher activation in the prefrontal cortex, especially in the frontal pole, could be associated with monitoring the expected outcome and putting on hold an alternative behavior during the current course of action (Koechlin, 2011).

Throughout this article, we have referred to the behavior of turning as associated with cooperating and the decision to continue as defection, following established conceptualizations in previous literature (see e.g., de Heus et al., 2010; Rapoport & Chammah, 1966; Wang et al., 2021). However, it is crucial to acknowledge that deciding on the noncompetitive alternative (to turn) might underlie several factors including risk avoidance (i.e., avoiding car crashes) or conflict de‐escalation (Koch et al., 2024). Yet, turning behavior, particularly in the context of turn‐taking strategy, is predominantly associated with cooperative behavior as indicated by a positive association between turn‐taking strategy and perceived coordination and fairness. Additionally, our previous behavioral study utilizing the Interactive Chicken Game task with another sample revealed that sibling pairs with low dominance traits and more supportive and cooperative relationship used this strategy more frequently (Hernandez‐Pena et al., 2023). Considering alternative influence factors, turning behavior cannot be definitively attributed to cooperative motives in every trial, but we affirm that turn‐taking behavior is plausibly linked with cooperation.

### 4.4 Sex differences and sibling relationship

In line with our hypotheses, there were sex differences in sibling relationship. Sisters reported closer and more supportive bonds, whereas brothers described a more apathetic and conflictual relationship, similar to prior studies (Hernandez‐Pena et al., 2023; Jensen et al., 2023; Salmon & Hehman, 2015; Stocker et al., 2020). This relates to higher cooperative behavior (use of turn‐taking strategy more frequently) among sisters, consistent with our previous behavioral study using the Interactive Chicken Game task (Hernandez‐Pena et al., 2023). These findings contrast with prior literature indicating greater cooperation in male–male interactions (Balliet et al., 2011), but most studies have explored dyads of strangers. This could point in the direction that participants' relationship influences their behavior. However, our analysis revealed that only sex significantly predicted competitive behavior, neither sibling competition scores nor age difference.

Sex differences were observed not only at the behavioral level, but also at the brain level. Men, who behaved more competitively, were also associated with higher activity in the right TPJ during dynamic decision‐making. In an exploratory whole‐brain analysis, we also observed increased activation (especially in defection over cooperation in both static and dynamic decision‐making phases) in areas such as bilateral SMG, precuneus, and fusiform gyri, right hippocampus, and left thalamus, compared with women. These exploratory findings should be taken with caution and need further replication. These results are in line with previous studies showing that men exhibit higher TPJ, SFG, and thalamic activity during mentalizing and PD tasks (Gao et al., 2019; Krach et al., 2009; Veroude et al., 2016). Others have interpreted an increased brain activation in men as compensation for their poorer mentalizing abilities (Gao et al., 2019; Krach et al., 2009). This compensatory “effect” may explain part of our results, in which men may need higher activation to predict other's behavior, especially in the dynamic phase, where we observed the sex differences. Furthermore, we propose that sisters, who have a closer relationship, might not need a deep mentalizing process as they might know more accurately their sister's general pattern and intentions. Therefore, the level of uncertainty may be lower which would require less theory of mind engagement (Rusch et al., 2020). We found only weak evidence for a direct connection between participants' relationship and task behavior or brain activation patterns and exclusively in the context of dynamic decision‐making. In prior literature, sibling intimacy in brother pairs has been associated with risky decision‐making (Solmeyer et al., 2014). In non‐risky decision‐making, sibling closeness was positively associated with activity in the right precentral gyrus (Rogers et al., 2018). Siblings also influenced externalizing behavior, but only when pairs were close in age (Trim et al., 2006). We did not find that age difference was a positive prediction for competitive behavior or brain activation during decision‐making. The sibling competition score was a significant predictor of brain activation in the right inferior and middle frontal gyri, and precuneus during cooperation in dynamic decision‐making, but it did not survive multiple comparison correction. A larger comparison in a more heterogeneous sample is needed. Further research should explore alternative explanatory factors for different decision‐making styles such as parenting styles (Davids et al., 2016), birth order (Rogers et al., 2018), or sibling's modeling perceptions (Rogers et al., 2021).

Our findings highlight the importance of a dynamic environment in investigating the brain correlates of mentalizing by using a “second‐person” approach to investigating neural correlates in real‐time ongoing social interactions. Future studies should use more interactive and ecological methods to fully investigate the role of mentalizing in social decision‐making. Also, exploring uncertainty levels across the different decision‐making phases by assessing participants' confidence in their intentions and predictions of the other's intentions, while considering individual and relationship factors, represents a promising direction for future studies. Subsequent research can delve into the neural patterns during the feedback phase comparing the different outcomes and feedback points, as well as exploring the inter‐brain activity and synchronization patterns between two simultaneously recorded brains (i.e., hyperscanning). Additionally, applying this task to clinical populations (e.g., depression, autism spectrum disorder, schizophrenia, or social anxiety disorder) with disrupted decision‐making (Báez‐Mendoza et al., 2021; Robson et al., 2020) may provide insights into specific challenges during the different decision‐making phases.

### 4.5 Limitations

The present study has several limitations that should be addressed in future work. First, compared with previous game theory paradigms, we were unable to explore brain activation underlying the different outcomes (mutual defection, mutual cooperation, and unilateral defection/cooperation) since we studied naturally occurring decisions and, therefore, we do not have an equal number of occurrences per pair. Second, we did not include a social value orientation questionnaire as previously done in other studies (Emonds et al., 2011), which could provide further explanations comparing prosocial and proself individuals. Third, we only included same‐sex sibling pairs because of time and funding constraints of this study and no nonsibling control group, which limits the generalizability of the results. The main reasons we did not include nonsibling pairs were (a) sample size, which would require measuring an additional 100 people, and (b) the difficulty in controlling for some characteristics that might explain participants' behavior, such as co‐residence of at least 10 years or shared genetics. Future studies are warranted that replicate our findings and explore further if there is something unique in siblings' cooperative and competitive interactions.

### 4.6 Conclusion

Our study aimed to authentically mirror real‐life social interactions between familiar individuals, by highlighting the dynamic nature of continuous real‐time decision‐making compared with traditional static game theory paradigms. Our findings revealed shared neural correlates involving mentalizing areas (TPJ, precuneus, and PFC) in both static and dynamic decision‐making phases. The static phase activated regions typically associated with action planning and outcome evaluation (including SFG and striatum) while the dynamic decision‐making phase in a changing environment engaged more strongly areas related to predicting others' intentions and monitoring their behavior (PFC, TPJ, ACC). Notably, cooperation (turning choice) during static decision‐making activated mentalizing‐related regions and brain areas associated with risk avoidance. On the other hand, defection (continuing choice) in the dynamic (action) phase triggered regions associated with conflict monitoring (ACC). Brothers displayed less cooperative behavior but showed higher TPJ activation during dynamic decision‐making. Sibling competition did not directly predict task behavior but showed a tendency to predict brain activation during interactive decision‐making. A broader and more flexible range of task behaviors allows for a deeper investigation of decision‐making processing. Our results underscore the significance of considering real interactions and the dynamic and interactive elements in the study of social decision‐making.

## AUTHOR CONTRIBUTIONS

L.H.‐P. wrote the original article. L.H.‐P. and L.W. designed the study. L.H.‐P. and R.S. programmed the tasks. L.H.‐P., J.K., R.S., and L.W. collected the data. L.H.‐P. and L.W. performed the data analysis and interpreted the data. L.H.‐P. visualized the data and designed the figures. L.H.‐P., J.K., E.B., J.S., A.M.‐L., R.W., U.H., R.S., and L.W. reviewed, edited, and approved the article.

## CONFLICT OF INTEREST STATEMENT

The authors declare that they have no known competing financial interests or personal relationships that could have appeared to influence the work reported in this article.

## Supporting information


**Data S1:** Supporting information.

## Data Availability

The study was preregistered at the open science framework (OSF; https://osf.io/5jbk8). The Interactive Chicken Game task adapted for hyperscanning fMRI measurements (PsychoPy3 version 2020.2.10) is available from the corresponding author upon request. Datasets and scripts are available on the OSF project (https://osf.io/zhwmu/). Group‐level maps from GLM analysis are available on the NeuroVault website (https://identifiers.org/neurovault.collection:15678).
