# Supplementary material for: Neural correlates of static and dynamic social decision‐making in real‐time sibling interactions
Source: Hum Brain Mapp. 2024 Jul 19;45(11):e26788. doi: 10.1002/hbm.26788 (PMC11258888; doi:10.1002/hbm.26788)
Supplement: Supplementary file 1 — Data S1: Supporting information. [file HBM-45-e26788-s001.docx]

**Supplementary Material: Neural correlates of static and dynamic social decision-making in real-time sibling interactions.**

Lucia Hernandez-Pena^1,2*^, Julia Koch^1,2^, Edda Bilek^3,4^, Julia Schräder^1,2^, Andreas Meyer-Lindenberg^4^, Rebecca Waller^5^, Ute Habel^1,2^, Rik Sijben^6^, Lisa Wagels^1,2^

^1^ Department of Psychiatry, Psychotherapy and Psychosomatics, Faculty of Medicine, RWTH Aachen, Pauwelsstrasse 30, 52074 Aachen, Germany.

^2^ Institute of Neuroscience and Medicine: JARA‑Institute Brain Structure Function Relationship (INM 10), Research Center Jülich, Jülich, Germany.

^3^ Wellcome Centre for Human Neuroimaging, Institute of Neurology, University College London, 12 Queen Square, London WC1N 3AR, United Kingdom.

^4^ Department of Psychiatry and Psychotherapy, Central Institute of Mental Health, Medical Faculty Mannheim, Heidelberg University, Mannheim, Germany.

^5^Department of Psychology, University of Pennsylvania, Philadelphia, PA, USA.

^6^ Brain Imaging Facility, Interdisciplinary Center for Clinical Research (IZKF), RWTH Aachen University, Aachen, Germany.

* Corresponding author: Lucia Hernandez-Pena

Department of Psychiatry, Psychotherapy and Psychosomatics, Faculty of Medicine, RWTH Aachen, Pauwelsstrasse 30, 52074 Aachen, Germany; Phone: +49 241 80-85333;

E-mail: [lhernandez@ukaachen.de](mailto:lhernandez@ukaachen.de)

1. **Demographics**

Brothers (*M* = 22.27, *SD* = 3.18) did not significantly differ in age compared to sisters (*M* = 21.85, *SD* = 2.38), *t*(88.62) = .728, *p* = .469. Brother pairs (*M* = 28.55, *SD* = 14.05) did not significantly differ in age difference in months compared to sisters (*M* = 28.37, *SD* = 14.68), *t*(93) = .062, *p* = .951.

**Supplementary Table S1.** Sociodemographic characteristics of the entire sample (*N* = 95), and separated by sex.

| Characteristic | Total  *N* = 95 | | Men  *n* = 49 | | Women  *n* = 46 | | Pearson’s chi-squared test | | | | Fisher’s exact test | |
| --- | --- | --- | --- | --- | --- | --- | --- | --- | --- | --- | --- | --- |
|  | *N* | % | *n* | % | *n* | % | *X^2^* | *df* | *p* | *P* | |  |
| **Handedness** |  |  |  |  |  |  | .009^a^ | 1 | .926 | 1.000 | |  |
| Right-handed | 87 | 91.58 | 45 | 91.84 | 42 | 91.30 |  |  |  |  | |  |
| Left-handed | 8 | 8.42 | 4 | 8.16 | 4 | 8.70 |  |  |  |  | |  |
| **Marital status** |  |  |  |  |  |  | 1.101^a^ | 2 | .577 | .915 | |  |
| Single | 53 | 55.79 | 28 | 57.14 | 25 | 54.35 |  |  |  |  | |  |
| In a relationship | 41 | 43.16 | 21 | 42.86 | 20 | 43.48 |  |  |  |  | |  |
| Married | 1 | 1.05 | 0 |  | 1 | 2.17 |  |  |  |  | |  |
| **Employment** |  |  |  |  |  |  | 4.74^a^ | 3 | .192 | .228 | |  |
| Student | 44 | 46.32 | 19 | 38.78 | 25 | 54.35 |  |  |  |  | |  |
| Unemployed/seeking work | 0 |  | 0 |  | 0 |  |  |  |  |  | |  |
| Part-time employed | 3 | 3.16 | 1 | 2.04 | 2 | 4.35 |  |  |  |  | |  |
| Full-time employed | 10 | 10.53 | 7 | 14.29 | 3 | 6.52 |  |  |  |  | |  |
| Not specified or missing data | 38 | 39.99 | 22 | 44.90 | 16 | 65.22 |  |  |  |  | |  |
| **Highest educational level** |  |  |  |  |  |  | 8.98^a^ | 5 | .110 | .069 | |  |
| Middle School | 2 | 2.11 | 2 | 4.08 | 0 |  |  |  |  |  | |  |
| Apprenticeship | 1 | 1.05 | 0 |  | 1 | 2.17 |  |  |  |  | |  |
| Specialized baccalaureate (High School) | 3 | 3.16 | 1 | 2.04 | 2 | 4.35 |  |  |  |  | |  |
| Specialized university exam (High School) | 62 | 65.26 | 30 | 61.22 | 32 | 69.57 |  |  |  |  | |  |
| University | 24 | 25.26 | 16 | 32.65 | 8 | 17.39 |  |  |  |  | |  |
| Other | 3 | 3.16 | 0 |  | 3 | 6.52 |  |  |  |  | |  |
| **Monthly household budget** |  |  |  |  |  |  | 1.563^a^ | 5 | .906 | .919 | |  |
| < 1,050 € | 42 | 44.21 | 21 | 42.86 | 21 | 45.65 |  |  |  |  | |  |
| 1,050 € to 1,410 € | 7 | 7.37 | 3 | 6.12 | 4 | 8.70 |  |  |  |  | |  |
| 1,410 € to 2,640 € | 10 | 10.53 | 6 | 12.24 | 4 | 8.70 |  |  |  |  | |  |
| 2,640 € to 4,400 € | 5 | 5.26 | 2 | 4.08 | 3 | 6.52 |  |  |  |  | |  |
| > 4,400 € | 3 | 3.16 | 2 | 4.08 | 1 | 2.17 |  |  |  |  | |  |
| Not specified or missing data | 28 | 29.47 | 15 | 30.61 | 13 | 28.26 |  |  |  |  | |  |

*Note*. ^a^ More than 20% of cells have an expected count of less than 5 and, therefore, Fisher’s exact test was considered instead (Kim, 2017).

### **Interactive Chicken Game questions**

After each block, participants in the Interactive Chicken Game task were asked to answer questions about perceived responsibility for the game outcome, motivation, and ideas and feelings about winning and losing. Further, feedback was presented indicating whether the player had won, lost, or tied the block (based on the comparison of the sum of the points that each participant gained or lost over the trials in the block). After completing the task, participants were asked about their own and their sibling's anticipated game intentions and strategies. See Supplementary Table S2 for a detailed explanation of the questions.

### **Supplementary Table S2.** Interactive Chicken Game Questions.

| **Time point** | **Variable** | **Question/Item** | **Response options** |
| --- | --- | --- | --- |
| Between each block | Outcome responsibility | Which of you was more responsible for the outcome? | Index finger = especially me  Middle finger = both equally  Ring finger = especially my sibling |
|  | Motivation | In the last block, I had the following thoughts: | 0 = “I didn't care who won.”  (0,10,20,30,40,50,60,70,80,90,100)  100 = “I wanted to win.” |
|  | Winning question  Powerful  Superior  Afraid | Please rate HOW YOU FELT after you WON a trial:  Use the following scale:  I felt powerful.  I felt superior.  I was afraid of revenge.  I felt happy.  I felt guilty.  I had the feeling of being dominant.  I had the feeling of being fair. | 1 = Not at all  2 = A little  3 = Neutral  4 = Considerably  5 = Extremely |
|  | Happy |  |  |
|  | Guilty |  |  |
|  | Dominant |  |  |
|  |  |  |  |
|  | Fair |  |  |
|  |  |  |  |
|  |  |  |  |
|  | Losing question  Failure | Please rate HOW YOU FELT after you LOST a trial:  Use the following scale:  I felt like  I felt like a failure.  I felt inferior.  I felt angry.  I felt vengeful.  I felt happy for my brother/sister.  I felt sad.  I felt it served me right. | 1 = Not at all  2 = A little  3 = Neutral  4 = Considerably  5 = Extremely |
|  | Inferior |  |  |
|  | Angry |  |  |
|  | Vengeful |  |  |
|  | Happy for other |  |  |
|  |  |  |  |
|  | Sad |  |  |
|  | Deserved |  |  |
| End of the task | Decision phase  Behavior expected | When do you think you mostly made the decision during the game?  Do you think your sibling behaved as you expected? | Single choice:  Index finger = When I saw the feedback  Middle finger = At the first question (turn/continue)  Ring finger = while driving  Little finger = different  1= Always  2 = Most of the time  3 = Half of the time  4 = Sometimes  5 = Never |
|  | Statements | To what extent do you think the following statements describe the game so far? | 1 = A little  (1,2,3,4,5)  5 = A lot |
|  | Sibling dominant | My brother/sister constantly tries to be in charge of the game. |  |
|  | Sibling aggressive | My brother/sister tries to harm me by not swerving, even if it leads to an accident. |  |
|  | Participant subordinate | I do not dare to drive on. |  |
|  | Perception of fairness | If he/she treats me fairly, I'll be fair too! |  |
|  | Sibling subordinate | My brother/sister does not dare to drive through. |  |
|  | Perception of coordination | My brother/sister and I try to coordinate to make similar gains. |  |
|  | Participant aggressive | I just want to harm my brother/sister. |  |
|  | Participant dominant | I’m dominating the game. |  |
|  | Perception of turn-taking | I adapt to the strategy of my brother/sister. |  |
|  | Strategy | Which strategy did you use most of the time? | single choice:   - I repeated what my brother/sister did in the last round. - If I won, I did the same thing again, otherwise, I changed my strategy. - I tried to help my brother/sister no matter how he/she acted. - I tried to harm my brother/sister no matter how he/she acted. - I tried to be the superior one, regardless of my brother/sister's behavior. - I tried to play as fair as possible. |

*Note.* These questions/items and response options were originally created and validated for the Interactive Chicken Game task (Hernandez-Pena et al., 2023). A German version of these questions was used in our study.

1. **Effect of time analysis**

To explore the effect of time in our task, we conducted Friedman tests, including block number (three levels) as the independent factor, and the number of conditions (one-turning, both turning, and both crashing) per block and per pair as dependent variables. Post-hoc tests were performed using Dunn´s test. These analyses were calculated on within-dyad scores as the outcome of each trial depended on the behavior of both participants. Using the individual data, a Friedman test was calculated, including block (three levels) as the independent factor, and the reaction time (RT) when turning as dependent variable. Finally, we used Friedman tests to explore the effect of time in the winning and losing questions between blocks across all participants (see supplementary Table S2 for further description of the questions). Block was included as independent variable and questions scores as dependent variables. We corrected for multiple comparisons for the winning (corrected α = .007) and losing (corrected α = .007) questions.

1. **Behavioral results**

There was a significant, but small, difference in the frequency of cooperation and defection trials comparing static (45.89% cooperative decisions) and dynamic (44.74% cooperative decisions) decision-making (X^2^(1, 95) = 1534.94, *p* < .001), across all trials. Significant differences were also found within each phase on the distribution of choices, with more defection trials compared to cooperation trials in static decision-making (X^2^(1, 95) = 10.87, *p* = .001), and in dynamic decision-making (X^2^(1, 95) = 44.21, *p* < .001), see Supplementary Table S3 for further details.

**Supplementary Table S3.** Behaviour trial frequency (*n* = 3990 trials).

|  | Total |  |
| --- | --- | --- |
|  | *%* | |
| **Static decision-making^a^** |  | |
| Cooperation (turn) | 45.89% | |
| Defection (continue) | 50.93% | |
| Missing | 3.08% | |
| **Dynamic decision-making^a^** |  | |
| Cooperation (turn) | 44.74% | |
| Defection (continue) | 55.26% | |
| **Static decision-making outcome^b^** |  | |
| Both crashing (mutual defection) | 24.12% | |
| Both turning (mutual cooperation) | 20.39% | |
| One turning (unilateral cooperation) | 49.28% | |
| Missing | 6.31% | |
| **Dynamic decision-making outcome (feedback phase)^b^** |  | |
| Both crashing (mutual defection) | 22.10% | |
| Both turning (mutual cooperation) | 12.11% | |
| One turning (unilateral cooperation) | 65.79% | |

*Note*. ^a^ Scores were calculated with individual data (*N* = 95). ^b^ Scores were calculated based on pair data (*N* = 46 pairs).

Comparing the dyad’s outcomes between the two decision phases, the frequency of a dyad’s number of crashing trials (mutual defection) in static decision-making (*Mdn)* = 8.00, approx. 24%) and dynamic decision-making (*Mdn* = 6.50, approx. 22%) did not significantly differ (*z* = -1.22, *p* = .222). However, the frequency of both turning trials (mutual cooperation) in static decision-making (*Mdn* = 6.00, approx. 20%) was higher (*z* = 4.291, *p* < .001) than in dynamic decision-making (*Mdn* = 3.00, approx. 12%). Furthermore, the one-turning (unilateral cooperation/defection) condition was selected significantly lower in static decision-making (*Mdn* = 20.00, approx. 49%) than in dynamic decision-making (*Mdn* = 30.00, approx. 66%), (*z* = -5.525, *p* < .001), see Supplementary Table S3 and Fig. 3.a. for more details.

When examining the dyads’ outcomes within static decision-making, the number of unilateral cooperation/defection condition was significantly higher than the number of mutual defection trials (*z* = -4.03, *p* < .001) and mutual cooperation trials (*z* = -4.64, *p* < .001), see Fig. 3.a. The number of mutual defection trials did not significantly differ from the number of mutual cooperation trials (*z* = -1.15, *p* = .249). In dynamic decision-making, mutual cooperation was selected significantly less often than mutual defection (*z* = -2.75, *p* = .006), and unilateral cooperation/defection condition (*z* = -5.50, *p* < .001). Mutual defection was chosen significantly less often than unilateral cooperation/defection (*z* = -5.18, *p* < .001), see Figure 3.a. The results remained the same as in the previous analyses after excluding two extreme outliers in the mutual cooperation condition; see below for statistical details. In Supplementary Figure S1, the distribution of each condition in all pairs included in the study can be found.

**Outcome results without outliers** (*N* = 44 pairs)

After removing two extreme outliers in the mutual cooperation condition, all results remain the same. The frequency of both turning (mutual cooperation) in static decision-making (*Md* = 5.00) was higher (*z* = -4.171, *p* < .001) than in dynamic decision-making (*Md* = 3.00).


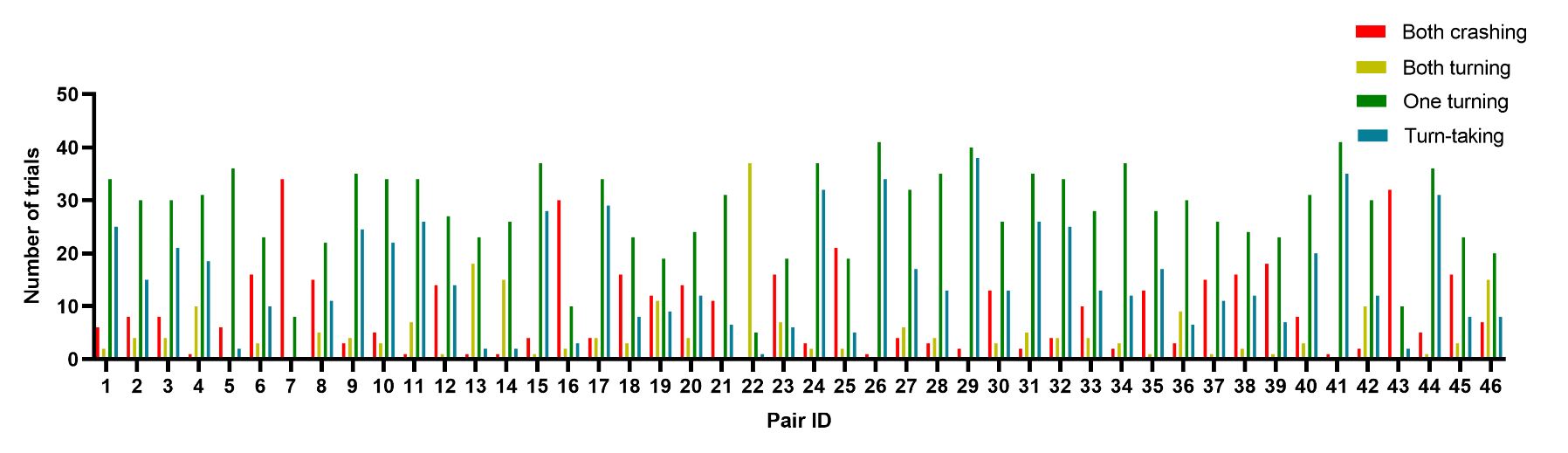
In static decision-making, mutual cooperation was chosen significantly less often than unilateral cooperation/defection condition (*z* = -5.05, *p* <.001). The frequency of mutual cooperation did not differ from mutual defection (*z* = .86, *p* = .391). In dynamic decision-making, mutual cooperation was selected less often than unilateral cooperation/defection condition (*z* = 5.78, *p* < .001) and mutual defection (*z* = 3.44, *p* = .001).

**Figure S1.** Bar charts depicting the distribution of each condition per pair during the Interactive Chicken Game task and showing the variability between pairs with some pairs mainly both turning, both crashing, or turn-taking. In the y-axis, the number of trials (*n* = 42 trials) per condition is represented. The x-axis represents the pair ID number.

Participants were significantly more consistent than inconsistent in their choices between static and dynamic decision-making phases during cooperation (z = -8.01, *p* < .001) as well as defection (z = -8.33, *p* < .001), see Figure 3.c and Supplementary Table S4 for further details. Comparing choice consistency between the two decision phases, there were significantly more consistent trials in defection than cooperation (z = -2.68, *p* = .007). There were no significant differences in inconsistency conditions comparing cooperation vs defection (z = -1.41, *p* = .159).

**Supplementary Table S4.** Interactive Chicken Game task behavior.

|  | Total  (*N* = 95; *N* = 46 pairs) | | Men  (*n* = 49, *n* = 24 pairs) | | | Women  (*n* = 46, *n* = 22 pairs) | | | Statistics | | | | |
| --- | --- | --- | --- | --- | --- | --- | --- | --- | --- | --- | --- | --- | --- |
|  | *M* | *SD* | *M* | *SD* | *M* | | *SD* | *df* | | *t* | *U* | *p* |  |
| **Outcome (feedback) ^a^** |  |  |  |  |  | |  |  | |  |  |  | |
| Both crashing (mutual defection) | 9.28 | 8.42 | 11.79 | 10.09 | 6.55 | | 5.04 |  | |  | 189.00 | .098 | |
| Both turning (mutual cooperation) | 4.87 | 6.42 | 5.83 | 7.85 | 3.82 | | 4.31 |  | |  | 218.50 | .313^c^ | |
| One turning (unilateral cooperation) | 27.85 | 8.60 | 24.38 | 9.36 | 31.64 | | 5.80 | 44 | | -3.13 |  | **.003** | |
| Turn-taking strategy | 15.07 | 10.32 | 11.54 | 9.51 | 18.91 | | 9.97 |  | |  | 136.00 | **.005** | |
| Dominance ICG score | 186.17 | 168.18 | 236.66 | 201.34 | 131.09 | | 100.60 |  | |  | 843.00 | **.034** | |
| **Consistency ^b^** |  |  |  |  |  | |  |  | |  |  |  | |
| Consistent cooperation  Consistent defection  Inconsistent cooperation  Inconsistent defection | 15.00  18.20  4.27  3.23 | 7.82  8.09  4.15  3.02 | 15.10  18.18  4.86  2.43 | 9.11  9.48  4.49  2.40 | 14.83  18.28  3.65  4.09 | | 6.35  6.49  3.71  3.38 |  | |  | 1105.00  1123.00  974.00  773.00 | .870  .976  .251  **.008** ^d^ | |
| **Reaction time ^b^** |  |  |  |  |  | |  |  | |  |  |  | |
| RT turning | 2.84 | 1.11 | 2.89 | 1.19 | 2.78 | | 1.03 | 93 | | .48 |  | .633 | |

*Note*. ^a^Scores were calculated based on pair data (*N* = 46 pairs). ^b^Scores were calculated with individual data (*N* = 95). RT = reaction time. Statistically significant values are shown in bold.

**Supplementary Table S5**. Correlations between behavior conditions and post-task self-reported game strategies *(n = 95)*

| **Variable** | **Both crashing** | **Both turning** | **Turn-taking** |
| --- | --- | --- | --- |
| Sibling dominant | .22 | -.11 | -.21 |
| Sibling aggressive | .56* | -.16 | -.19 |
| Sibling subordinate | -.12 | .05 | .04 |
| Participant dominant | .26 | -.16 | -.22 |
| Participant aggressive | .28 | -.03 | -.18 |
| Participant subordinate | -.11 | .07 | .14 |
| Perception fairness | -.34* | .15 | .36* |
| Perception coordination | -.28 | -.02 | .47* |
| Perception turn-taking | -.20 | .01 | .20 |

*Note.* *Significant after Bonferroni correction (corrected α = 0.0018). Consult Supplementary Table S2 for further description of the game strategy statements.

At the end of the completed task, participants were asked about the phase they usually made their decisions on across trials. There was a significant difference in the distribution of responses (X^2^(3, 94) = 23.19, *p* < .001). Significantly more participants indicated that they made their decisions during the static decision-making phase (44.2%), followed by during "different phases" (24.2%), and during dynamic decision-making (20.0%). A minority of participants chose the feedback phase to make their decisions (10.5%), missing data (1.1%).

**5. Effect of time results**

**5.1. Linear mixed model: trial-by-trial results**

The results of the linear mixed model analysis on RT in the static decision-making revealed that trial number significantly predicted RT (*F*(1, 3814.72) = 107.56, *p* < .001), with participants responding faster over time. Neither static decision-making choice (*F*(1, 3857.66) = .14, *p* = .709), nor dynamic decision-making choice (*F*(1, 3833.62) = 2.42, *p* = .120) were significant predictors of RT while answering the planning question in the static phase. For further details on the linear mixed model results, see Supplementary Table S6. However, the interaction between static and dynamic decision-making choices significantly predicted RT (*F*(1, 3838.79) = 5.60, *p* = .018). Specifically, incongruent conditions (different decisions in static and dynamic phases) exhibited a longer time for deciding in the static phase than congruent conditions; see below Supplementary Table S7.

**Supplementary Table S6.** Behavioral linear mixed models with RT as dependent variable *(n = 95)*

*Linear mixed model expression*: Static decision-making RT ~ static decision-making choice + dynamic decision-making choice + trial number + (1 | subject_ID)

|  | *Coefficient* | *S.E.* | *df* | *t* | *Sig.* | *95% CI*  *Lower bound Upper bound* | |
| --- | --- | --- | --- | --- | --- | --- | --- |
| **RT** |  |  |  |  |  |  |  |
| Intercept | 1.773 | .064 | 94.69 | 27.85 | **<.001** | 1.646 | 1.899 |
| Trial number | -.013 | .001 | 3814.72 | -10.37 | **<.001** | -.015 | -.010 |
| Static decision-making (continue) | -.079 | .059 | 3849.81 | -1.31 | .190 | -.039 | .196 |
| Dynamic decision-making (continue)  Interaction  (Static decision-making continue-Dynamic decision-making continue) | .033  -.187 | .053  .079 | 3837.54  3838.79 | .61  -2.37 | .540  **.018** | -.072  .0321 | -.137  .343 |

*Linear mixed model expression*: Dynamic decision-making RT ~ trial number + (1 | subject_ID)

|  | *Coefficient* | *S.E.* | *df* | *t* | *Sig.* | *95% CI*  *Lower bound Upper bound* | |
| --- | --- | --- | --- | --- | --- | --- | --- |
| **RT** |  |  |  |  |  |  |  |
| Intercept | 3.230 | .159 | 65.23 | 20.36 | **<.001** | 2.913 | 3.547 |
| Trial number | -.016 | .003 | 1739.50 | -5.96 | **<.001** | -.022 | -.011 |

**Supplementary Table S7**. Interaction effects in linear mixed model for static decision-making RT.

| Static decision-making choice | Dynamic decision-making choice | *Mean* | *S.E.* | *df* | *95% CI*  *Lower bound Upper bound* | |
| --- | --- | --- | --- | --- | --- | --- |
| Continue | Continue  Turn | 1.42  1.58 | .06  .07 | 58.12  173.45 | 1.310  1.431 | 1.535  1.724 |
| Turn | Continue  Turn | 1.53  1.50 | .07  .06 | 135.48  62.78 | 1.394  1.384 | 1.669  1.614 |

**5.2. Block wise results**

A significant effect of block was observed in both crashing (X^2^(2, 95) = 8.92, *p* = .012), with an increase in the number of both crashing trials across time (block 1 > block 2). Similarly, there was an effect of block in one-turning, (X^2^(2, 95) = 7.23, *p* = .027; block 2 > block 1). There was also a significant effect of block in both turning (X^2^(2, 95) = 6.86, *p* = .032), but pairwise comparisons between blocks were not significant (*p* > .05). After removing two extreme outliers, the block effect was no longer significant (X^2^(2, 93) = 4.42, *p* = .110). A significant block effect was also observed in RT when participants turned (X^2^(2, 87) = 15.31, *p* < .001), with the first block having significantly longer RTs compared to the second and third blocks, in line with the results from the trial-by-trial model. For detailed statistics and further information, refer to Supplementary Figure S2 and Supplementary Tables S8. There was a significant effect of block in winning and losing questions resulting in decreased feelings of superiority and empowerment (for further description of the questions see Supplementary Table S2 and consult the Supplementary Table S8 and Figure S3 for statistics and further details).

**Supplementary Table S8.** Effect of time during the Interactive Chicken Game task.

|  | Block 1 | | Block 2 | | Block 3 | | Statistics | |  |  | |  | |
| --- | --- | --- | --- | --- | --- | --- | --- | --- | --- | --- | --- | --- | --- |
|  |  | | | | | | | | Post-hoc tests | | | | |
|  |  |  |  |  |  |  |  |  | Block  1 vs 2 | | Block 1 vs 3 | | Block 2 vs 3 |
|  | *M* | *SD* | *M* | *SD* | *M* | *SD* | *X^2^*(2) | *p* | *Adjusted p* | *Adjusted p* | | *Adjusted p* | |
| **Outcome**^a^ |  |  |  |  |  |  |  |  |  |  | |  | |
| Both crashing | 3.57 | 3.03 | 2.78 | 3.74 | 2.93 | 3.26 | 8.92 | **.012** | **.032** | .098 | | .999 | |
| Both turning | 1.59 | 2.08 | 1.22 | 1.92 | 2.07 | 3.25 | 6.86 | **.032** | .182 | .999 | | .182 | |
| Both turning* | 1.26 | 1.14 | 1.00 | 1.43 | 1.33 | 1.63 | 4.42 | .110 |  |  | |  | |
| One turning | 4.42 | 1.56 | 5.00 | 1.88 | 4.50 | 1.85 | 7.23 | **.027** | **.043** | .999 | | .229 | |
| **RT**^b^ |  |  |  |  |  |  |  |  |  |  | |  | |
| RT turning** | 3.17 | 1.24 | 2.68 | 1.21 | 2.62 | 1.43 | 15.31 | **< .001** | **.005** | **.001** | | .999 | |
| **How did you feel after winning?**^b^ |  |  |  |  |  |  |  |  |  |  | |  | |
| Powerful | 2.13 | 1.10 | 1.86 | 1.08 | 1.68 | .959 | 14.29 | **.001** | .245 | **.020** | | .982 | |
| Superior | 2.03 | 1.07 | 1.82 | 1.03 | 1.64 | 1.01 | 10.22 | **.006** | .613 | .061 | | .879 | |
| Afraid | 1.71 | 1.13 | 1.55 | 0.99 | 1.40 | 0.83 | 7.38 | **.025** | .999 | .245 | | .982 | |
| Happy | 3.23 | 1.23 | 3.02 | 1.30 | 3.05 | 1.41 | 2.80 | .246 |  |  | |  | |
| Guilty | 1.97 | 1.21 | 1.59 | 0.87 | 1.51 | 0.97 | 24.01 | **< .001** | .081 | **.005** | | .999 | |
| Dominant | 1.89 | 1.02 | 1.71 | 1.03 | 1.66 | 0.94 | 6.91 | **.032** | .612 | .285 | | .999 | |
| Fair | 3.35 | 1.24 | 3.07 | 1.35 | 3.31 | 1.34 | 3.08 | .214 |  |  | |  | |
| **How did you feel after losing?**^b^ |  |  |  |  |  |  |  |  |  |  | |  | |
| Failure | 1.74 | 1.06 | 1.61 | 0.97 | 1.32 | 0.67 | 19.56 | **< .001** | .999 | **.022** | | .138 | |
| Inferior | 1.62 | 0.98 | 1.56 | 0.93 | 1.63 | 1.09 | 1.81 | .405 |  |  | |  | |
| Angry | 1.84 | 1.10 | 1.58 | 0.97 | 1.64 | 1.06 | 4.62 | .099 |  |  | |  | |
| Vengeful | 1.48 | 0.89 | 1.46 | 0.90 | 1.53 | 1.14 | 0.07 | .965 |  |  | |  | |
| Happy for other | 3.07 | 1.34 | 3.29 | 1.39 | 3.27 | 1.41 | 1.86 | .394 |  |  | |  | |
| Sad | 1.26 | 0.66 | 1.28 | 0.72 | 1.16 | 0.49 | 6.20 | **.045** | .999 | .999 | | .878 | |
| Deserved | 1.72 | 1.13 | 1.61 | 0.98 | 1.69 | 1.13 | 1.48 | .488 |  |  | |  | |

*Note*. ^a^Scores were calculated based on pair data (*N* = 46). ^b^Scores were calculated with individual data (*N* = 95). *Results after excluding three outliers (*N* = 43). ** *N* = 87 (without missing values). Friedman tests and Dunn’s multiple comparison tests were used to calculate the differences (adjusted *p*-values are reported). RT = reaction time. Statistically significant values are shown in bold.


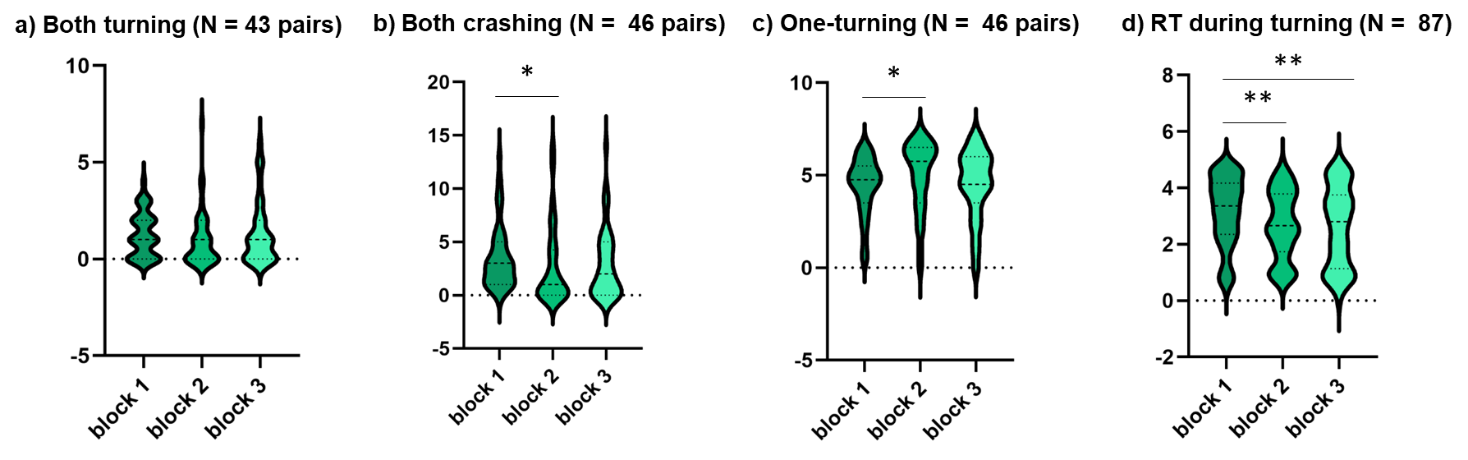


**Figure S2.** Violin plots depicting the distribution of each condition. The violin plots represent summary statistics and the kernel density estimation (y-axis) to show the frequency distribution of each condition. The dotted thick line in the middle represents the median, while the other thinner lines represent the quartiles. Friedman tests were used to calculate the effect of time on conditions. Significant differences between blocks are represented with asterisks: * *p* < .05, ** *p* < .01. a) both turning condition results only include 44 pairs after excluding two extreme outliers. b) both crashing condition results show a significant difference between block 1 > block 2. c) one-turning condition results show significant differences between block 2 > block 1. d) RT = reaction time when turning (based on individual data). Results show significant differences between block 1 > block 2 and block 1 > block 3.

**
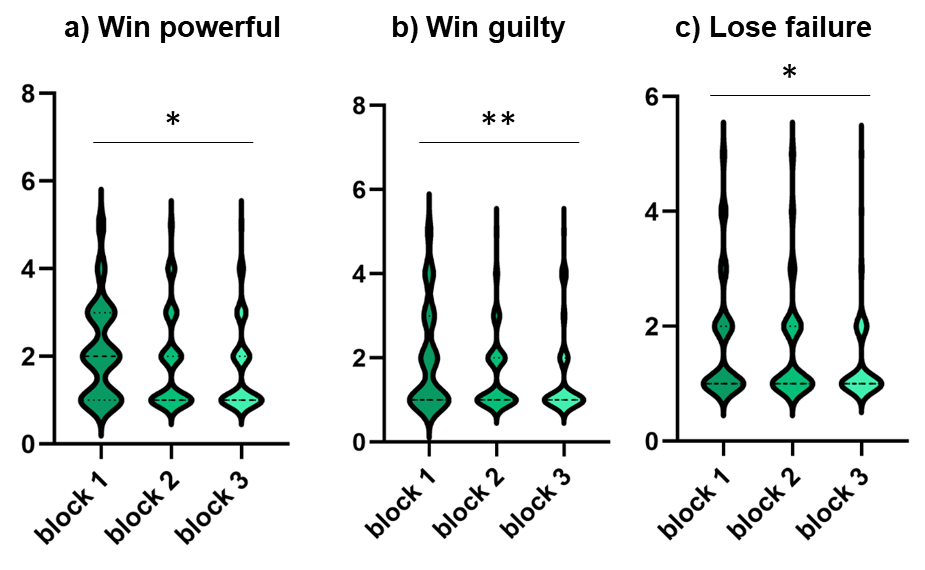
**

**Figure S3.** Violin plots depicting the distribution of each question (see Table S2 for question description) using individual data (*N* = 95). The violin plots represent summary statistics and the kernel density estimation (y-axis) to show the frequency distribution of each question. The dotted thick line in the middle represents the median, while the other thinner lines represent the quartiles. Friedman tests were used to calculate the effect of time on each question. Significant differences between blocks are represented with asterisks: * *p* < .05, ** *p* < .01.

**6. Effect of time discussion**

As expected, there was a learning effect over time, but in the opposite direction as hypothesized. In our study, participants exhibited more cooperative behavior over time, aligning with diminishing feelings of superiority, guilt, empowerment, and reduced perceptions of failure. The few studies analyzing the effect of time on the decision-making process found a decrease in cooperative response over time (Normann & Wallace, 2012; Ortmann & Tichy, 1999). However, relevant differences in the characteristics of the studies may underlie this inconsistency. Unlike previous studies using the Prisoner’s Dilemma, our study uses the Chicken Game task, which differs in the payoff matrix which could contribute to variations in cooperation rates (Kümmerli et al., 2007). Additionally, our study included siblings rather than strangers, potentially leading to increased cooperation due to the distinct nature of their relationship. While this interpretation remains speculative and lies beyond the scope of our current study design, it presents an intriguing prospect for future research. Regarding the decrease in reaction time in the static decision-making question, we speculate that it might be due to an effect of learning the sibling's intentions and establishing a firmer strategy throughout the task in which participants can more quickly estimate the other's intentions and make their own decisions. Previous literature has associated shorter decisions with more intuitive decisions compared to a control process (Achtziger et al., 2014). Thus, this could be supported by the finding that in trials in which participants change their minds (either they plan to defect and then turn in the dynamic phase or vice versa) it took longer to make a decision in the planning phase. This may imply a lack of confidence among participants regarding both their own and their siblings' strategies, manifesting in a longer duration to make these decisions and subsequent strategy changes. Although this raises a very interesting line of research, we do not have enough events of each condition to analyze the brain activity in each event. Future researchers should investigate this aspect further and determine whether incongruent trials are associated with increased activity in mentalizing-related brain regions.

**Supplementary Table S9.** Personality characteristics of the entire sample (*N* = 95) and separated by sex.

|  | Total | | Men | | Women | |  | | Statistics | | |
| --- | --- | --- | --- | --- | --- | --- | --- | --- | --- | --- | --- |
|  | *N* = 95 | | *n* = 49 | | *n* = 46 | |  |  | |  |  |
|  | *M* | *SD* | *M* | *SD* | *M* | *SD* | *df* | *t* | | *U* | *p* |
| **STQ** |  |  |  |  |  |  |  |  | |  |  |
| Mutuality^a^ | 46.44 | 7.76 | 44.06 | 7.35 | 48.98 | 7.44 | 93 | -3.24 | |  | **.002** |
| Competition^b^ | 12.92 | 4.66 | 12.69 | 4.39 | 13.17 | 4.97 |  |  | | 1055.00 | .591 |
| Criticism^b^ | 26.11 | 8.16 | 24.45 | 6.36 | 27.87 | 9.47 |  |  | | 903.00 | .095 |
| Apathy^b^ | 20.99 | 5.51 | 22.59 | 5.34 | 19.28 | 5.23 |  |  | | 698.00 | **.001** |
| Longing^b^ | 13.12 | 3.04 | 12.55 | 3.37 | 13.72 | 2.54 |  |  | | 839.00 | **.031** |
| **DoPL** |  |  |  |  |  |  |  |  | |  |  |
| Dominance^a^ | 25.67 | 6.31 | 26.98 | 6.69 | 24.28 | 5.62 | 93 | 2.12 | |  | **.037** |
| Prestige^b^ | 41.74 | 6.59 | 41.90 | 6.01 | 41.59 | 7.22 |  |  | | 1121.50 | .967 |
| Leadership^a^ | 36.43 | 6.82 | 37.63 | 6.77 | 35.15 | 6.71 | 93 | 1.79 | |  | .076 |
| **HCA**^b^ | 65.95 | 10.82 | 66.66 | 9.93 | 65.2 | 11.76 | 93 | .65 | |  | .514 |
| **PDCA**^b^ | 51.92 | 10.34 | 53.28 | 8.31 | 50.47 | 12.06 |  |  | | 989.50 | .305 |
| **RSPQ** |  |  |  |  |  |  |  |  | |  |  |
| DL^b^ | 18.24 | 2.84 | 18.90 | 2.76 | 17.53 | 2.77 |  |  | | 836.00 | **.029** |
| CB^b^ | 28.35 | 3.31 | 28.16 | 3.52 | 28.54 | 3.10 |  |  | | 1065.00 | .645 |
| RSA^b^ | 12.02 | 2.92 | 12.50 | 2.92 | 11.50 | 2.86 |  |  | | 868.50 | .053 |
| **SoPS**^b^ | 4.34 | 0.50 | 4.42 | 0.46 | 4.27 | 0.53 |  |  | | 928.00 | .137 |
| **BPAQ**^a^ | 62.09 | 9.15 | 62.79 | 9.74 | 61.35 | 8.52 | 93 | .77 | |  | .446 |
| **Mach-IV**^a^ | 51.40 | 7.47 | 52.87 | 7.12 | 49.84 | 7.60 | 93 | 2.01 | |  | **.047** |
| **BIS-11**^a^ | 59.78 | 8.76 | 61.79 | 8.92 | 57.63 | 8.13 | 93 | 2.37 | |  | **.020** |

*Note*. ^a^Independent sample t-test was used. ^b^Mann-Whitney U test was used because variables were not normally distributed. Statistically significant tests are highlighted in bold. STQ = Sibling Type Questionnaire (Stewart et al., 2001), DoPL = Dominance, Prestige and Leadership Motives scale (Suessenbach et al., 2019), HCA = Hypercompetitive Attitudes Scale (Ryckman et al., 1990), PDCA = Personal Development Competitive Attitudes Scale (Ryckman et al., 1996), RSPQ = Rank Style with Peers Questionnaire (RSPQ; Zuroff et al., 2010), DL = Dominant Leadership, CB = Coalition Building, RSA = Ruthless Self-Advancement, SoPS = Sense of Power Scale (Anderson et al., 2012), Buss-Perry Aggression Questionnaire (Buss & Perry, 1992), Mach-IV = Machiavellianism scale (Mach IV; Christie & Geis, 1970) and BIS-11 = Barratt Impulsiveness Scale (Patton et al., 1995).

**Supplementary Table S10.** Behavioral and brain Region of Interest (ROI) linear mixed model results.

*Linear mixed model expression*: ICG score ~ sex + sibling competition STQ z-score + age difference + (1 | pair_ID)

*Linear mixed model expression*: ROI ~ sex + sibling competition STQ z-score + age difference + (1 | pair_ID)

| Contrast |  | *Coefficient* | *S.E.* | *df* | *t* | *Sig.* | *95% CI*  *Lower bound Upper bound* | |
| --- | --- | --- | --- | --- | --- | --- | --- | --- |
| **Behavior analysis** | **ICG score** |  |  |  |  |  |  |  |
|  | Intercept | 50.725 | 29.997 | 43.06 | 1.69 | .098 | -9.768 | 111.218 |
|  | Sex | 52.37 | 23.941 | 43.83 | 2.19 | **.034** | 4.080 | 100.653 |
|  | STQ comp | -3.73 | 7.326 | 71.30 | -.51 | .612 | -18.336 | 10.878 |
|  | Age difference | .526 | .851 | 42.99 | .62 | .540 | -1.190 | 2.242 |
| **Static decision-making cooperation** | **Right IFG** |  |  |  |  |  |  |  |
|  | Intercept | .511 | .168 | 42.75 | 3.05 | .004 | .173 | .848 |
|  | Sex | .197 | .133 | 42.48 | 1.48 | .146 | -.072 | .466 |
|  | STQ comp | .126 | .067 | 77.69 | 1.88 | .064 | -.008 | .259 |
|  | Age difference | .005 | .005 | 42.67 | 1.15 | .257 | -.004 | .015 |
|  | **Left insula** |  |  |  |  |  |  |  |
|  | Intercept | .412 | .174 | 42.82 | 2.37 | .023 | .061 | .764 |
|  | Sex | .191 | .139 | 42.53 | 1.38 | .176 | -.089 | .471 |
|  | STQ comp | .080 | .066 | 84.08 | 1.22 | .227 | -.051 | .211 |
|  | Age difference | .002 | .005 | 42.73 | .34 | .737 | -.008 | .012 |
|  | **Right insula** |  |  |  |  |  |  |  |
|  | Intercept | .555 | .158 | 43.21 | 3.51 | .001 | .236 | .875 |
|  | Sex | .185 | .126 | 42.91 | 1.47 | .149 | -.069 | .440 |
|  | STQ comp | .039 | .057 | 86.81 | .68 | .501 | -.075 | .153 |
|  | Age difference | .001 | .004 | 43.11 | .11 | .910 | -.009 | .010 |
|  | **Right TPJ** |  |  |  |  |  |  |  |
|  | Intercept | 1.164 | .333 | 42.82 | 3.50 | .001 | .493 | 1.836 |
|  | Sex | .523 | .265 | 42.53 | 1.97 | .055 | -.012 | 1.057 |
|  | STQ comp | .009 | .129 | 81.82 | .07 | .947 | -.248 | .265 |
|  | Age difference | -.005 | .009 | 42.73 | -.55 | .584 | -.024 | .014 |
|  | **Right MFG** |  |  |  |  |  |  |  |
|  | Intercept | .214 | .338 | 43.16 | .63 | .529 | -.467 | .895 |
|  | Sex | .109 | .269 | 42.86 | .41 | .687 | -.433 | .651 |
|  | STQ comp | -.045 | .127 | 84.68 | -.35 | .725 | -.297 | .207 |
|  | Age difference | .020 | .010 | 43.06 | 2.07 | **.044** | .001 | .039 |
|  | **Right precuneus** |  |  |  |  |  |  |  |
|  | Intercept | .985 | .302 | 43.01 | 3.26 | .002 | .375 | 1.594 |
|  | Sex | -.022 | .240 | 42.74 | -.09 | .926 | -.507 | .463 |
|  | STQ comp | .052 | .121 | 77.65 | .43 | .667 | -.189 | .293 |
|  | Age difference | -.005 | .009 | 42.92 | -.55 | .585 | -.022 | .013 |
|  | **Right SFG** |  |  |  |  |  |  |  |
|  | Intercept | .383 | .255 | 42.58 | 1.50 | .140 | -.131 | .897 |
|  | Sex | .331 | .203 | 42.29 | 1.63 | .111 | -.079 | .740 |
|  | STQ comp | -.019 | .097 | 83.52 | -.20 | .844 | -.212 | .174 |
|  | Age difference | .007 | .007 | 42.49 | .96 | .342 | -.008 | .022 |
| **Static decision-making defection** | **Right IFG** |  |  |  |  |  |  |  |
|  | Intercept | .424 | .188 | 42.09 | 2.26 | .029 | .045 | .804 |
|  | Sex | .188 | .150 | 41.82 | 1.26 | .215 | -.114 | .490 |
|  | STQ comp | .129 | .076 | 76.76 | 1.70 | .093 | -.022 | .279 |
|  | Age difference | .003 | .005 | 42.01 | .60 | .549 | -.008 | .014 |
|  | **Left insula** |  |  |  |  |  |  |  |
|  | Intercept | .552 | .221 | 43.03 | 2.50 | .016 | .106 | .997 |
|  | Sex | .163 | .176 | 42.73 | .93 | .360 | -.192 | .518 |
|  | STQ comp | .100 | .085 | 82.88 | 1.18 | .241 | -.068 | .268 |
|  | Age difference | -.003 | .006 | 42.93 | -.52 | .606 | -.016 | .009 |
|  | **Right insula** |  |  |  |  |  |  |  |
|  | Intercept | .651 | .162 | 43.02 | 4.02 | .000 | .324 | .977 |
|  | Sex | .096 | .129 | 42.74 | .75 | .460 | -.164 | .356 |
|  | STQ comp | .100 | .064 | 79.35 | 1.56 | .122 | -.027 | .228 |
|  | Age difference | -.001 | .005 | 42.93 | -.19 | .849 | -.010 | .008 |
|  | **Right TPJ** |  |  |  |  |  |  |  |
|  | Intercept | .807 | .303 | 43.02 | 2.66 | .011 | .195 | 1.418 |
|  | Sex | .481 | .241 | 42.75 | 1.99 | .053 | -.006 | .967 |
|  | STQ comp | .047 | .121 | 78.10 | .39 | .696 | -.194 | .289 |
|  | Age difference | -.004 | .009 | 42.93 | -.45 | .654 | -.021 | .013 |
|  | **Right MFG^1^*** |  |  |  |  |  |  |  |
|  | Intercept | .163 | .322 | 86.00 | .51 | .613 | -.477 | .804 |
|  | Sex | .195 | .259 | 86.00 | .75 | .453 | -.319 | .709 |
|  | STQ comp | -.075 | .132 | 86.00 | -.57 | .572 | -.338 | .188 |
|  | Age difference | .014 | .009 | 86.00 | 1.51 | .136 | -.004 | .032 |
|  | **Right precuneus** |  |  |  |  |  |  |  |
|  | Intercept | .802 | .286 | 42.99 | 2.80 | .008 | .225 | 1.379 |
|  | Sex | .031 | .228 | 42.73 | .13 | .894 | -.429 | .490 |
|  | STQ comp | .047 | .115 | 77.14 | .41 | .683 | -.182 | .276 |
|  | Age difference | -.008 | .008 | 42.91 | -1.03 | .309 | -.025 | .008 |
|  | **Right SFG** |  |  |  |  |  |  |  |
|  | Intercept | .103 | .298 | 41.90 | .34 | .732 | -.499 | .704 |
|  | Sex | .364 | .237 | 41.62 | 1.54 | .132 | -.114 | .843 |
|  | STQ comp | .070 | .118 | 79.33 | .59 | .555 | -.164 | .304 |
|  | Age difference | .008 | .008 | 41.81 | 1.00 | .322 | -.009 | .026 |
| **Dynamic decision-making cooperation** | **Right IFG** |  |  |  |  |  |  |  |
|  | Intercept | .726 | .211 | 43.11 | 3.45 | .001 | .301 | 1.151 |
|  | Sex | .219 | .168 | 42.80 | 1.31 | .198 | -.119 | .558 |
|  | STQ comp | .174 | .075 | 87.63 | 2.34 | **.022** | .026 | .323 |
|  | Age difference | -.004 | .006 | 43.01 | -.68 | .501 | -.016 | .008 |
|  | **Left insula^2^** |  |  |  |  |  |  |  |
|  | Intercept | .547 | .222 | 42.16 | 2.46 | .018 | .099 | .996 |
|  | Sex | .118 | .179 | 41.95 | .66 | .513 | -.244 | .481 |
|  | STQ comp | .004 | .077 | 86.00 | .05 | .963 | -.149 | .156 |
|  | Age difference | -.003 | .006 | 41.92 | -.45 | .656 | -.016 | .010 |
|  | **Right insula** |  |  |  |  |  |  |  |
|  | Intercept | .526 | .198 | 43.31 | 2.65 | .011 | .126 | .926 |
|  | Sex | .145 | .158 | 43.00 | .92 | .362 | -.173 | .464 |
|  | STQ comp | .082 | .065 | 87.59 | 1.25 | .214 | -.048 | .212 |
|  | Age difference | -.003 | .006 | 43.21 | -.51 | .613 | -.014 | .008 |
|  | **Right TPJ^3^** |  |  |  |  |  |  |  |
|  | Intercept | .639 | .295 | 4.87 | 2.17 | .036 | .044 | 1.235 |
|  | Sex | .672 | .237 | 4.60 | 2.83 | **.007** | .193 | 1.151 |
|  | STQ comp | .000 | .112 | 81.38 | .00 | .997 | -.224 | .223 |
|  | Age difference | -.012 | .008 | 4.76 | -1.45 | .156 | -.029 | .005 |
|  | **Right MFG** |  |  |  |  |  |  |  |
|  | Intercept | .756 | .391 | 43.24 | 1.93 | .060 | -.033 | 1.545 |
|  | Sex | .732 | .312 | 42.93 | 2.35 | **.023** | .104 | 1.361 |
|  | STQ comp | .270 | .130 | 87.72 | 2.07 | **.041** | .011 | .528 |
|  | Age difference | -.002 | .011 | 43.14 | -.19 | .847 | -.025 | .020 |
|  | **Right precuneus*** |  |  |  |  |  |  |  |
|  | Intercept | .750 | .254 | 88.00 | 2.95 | .004 | -.033 | 1.545 |
|  | Sex | .235 | .202 | 88.00 | 1.16 | .248 | .104 | 1.361 |
|  | STQ comp | .224 | .103 | 88.00 | 2.19 | **.031** | .011 | .528 |
|  | Age difference | -.013 | .007 | 88.00 | -1.81 | .074 | -.025 | .020 |
|  | **Right SFG** |  |  |  |  |  |  |  |
|  | Intercept | .281 | .299 | 43.00 | .94 | .354 | -.323 | .885 |
|  | Sex | .559 | .238 | 42.69 | 2.34 | **.024** | .078 | 1.040 |
|  | STQ comp | .160 | .109 | 86.40 | 1.47 | .146 | -.057 | .378 |
|  | Age difference | .003 | .008 | 42.90 | .34 | .733 | -.014 | .020 |
| **Dynamic decision-making defection** | **Right IFG** |  |  |  |  |  |  |  |
|  | Intercept | .618 | .197 | 42.35 | 3.14 | .003 | .221 | 1.014 |
|  | Sex | .221 | .157 | 42.04 | 1.41 | .166 | -.095 | .537 |
|  | STQ comp | .115 | .070 | 87.33 | 1.64 | .105 | -.024 | .255 |
|  | Age difference | -.004 | .006 | 42.25 | -.67 | .506 | -.015 | .008 |
|  | **Left insula^4^** |  |  |  |  |  |  |  |
|  | Intercept | .586 | .181 | 42.00 | 3.23 | .002 | .220 | .952 |
|  | Sex | .201 | .146 | 41.66 | 1.38 | .174 | -.093 | .495 |
|  | STQ comp | -.057 | .068 | 84.02 | -.84 | .402 | -.193 | .078 |
|  | Age difference | -.005 | .005 | 41.90 | -1.06 | .295 | -.016 | .005 |
|  | **Right insula^5^** |  |  |  |  |  |  |  |
|  | Intercept | .518 | .139 | 41.20 | 3.72 | .001 | .237 | .799 |
|  | Sex | .236 | .112 | 4.85 | 2.11 | **.041** | .010 | .462 |
|  | STQ comp | .013 | .051 | 85.09 | .25 | .803 | -.089 | .115 |
|  | Age difference | -.003 | .004 | 41.09 | -.75 | .458 | -.011 | .005 |
|  | **Right TPJ** |  |  |  |  |  |  |  |
|  | Intercept | .247 | .258 | 42.15 | .96 | .345 | -.275 | .767 |
|  | Sex | .618 | .207 | 41.81 | 2.98 | **.005** | .200 | 1.037 |
|  | STQ comp | .079 | .093 | 85.66 | .85 | .397 | -.106 | .265 |
|  | Age difference | .001 | .007 | 42.04 | .06 | .956 | -.014 | .015 |
|  | **Right MFG** |  |  |  |  |  |  |  |
|  | Intercept | .667 | .288 | 42.53 | 2.31 | .026 | .086 | 1.248 |
|  | Sex | .325 | .229 | 42.24 | 1.42 | .164 | -.138 | .788 |
|  | STQ comp | .027 | .111 | 81.89 | .24 | .808 | -.194 | .249 |
|  | Age difference | .003 | .008 | 42.43 | .37 | .712 | -.013 | .020 |
|  | **Right precuneus^6^*** |  |  |  |  |  |  |  |
|  | Intercept | .706 | .195 | 86.00 | 3.62 | .000 | .318 | 1.094 |
|  | Sex | .118 | .157 | 86.00 | .75 | .454 | -.193 | .429 |
|  | STQ comp | .082 | .080 | 86.00 | 1.02 | .309 | -.077 | .241 |
|  | Age difference | -.011 | .006 | 86.00 | -1.92 | .059 | -.022 | .000 |
|  | **Right SFG** |  |  |  |  |  |  |  |
|  | Intercept | .522 | .244 | 41.28 | 2.14 | .038 | .030 | 1.014 |
|  | Sex | .380 | .194 | 41.00 | 1.96 | .057 | -.012 | .772 |
|  | STQ comp | -.005 | .096 | 79.86 | -.05 | .960 | -.195 | .185 |
|  | Age difference | .001 | .007 | 41.19 | .08 | .933 | -.013 | .015 |

*Note*: STQ comp = Competition subscale from the Sibling Type Questionnaire, IFG = inferior frontal gyrus, TPJ = temporo-parietal junction, MFG = middle frontal gyrus, SFG = superior frontal gyrus. Statistically significant values are shown in bold. All analyses were performed with all completed pairs (N = 94), except the following analysis due to outliers removals: **^1^***N* = 92, **^2^***N* = 92, **^3^***N* = 90, **^4^***N* = 92, **^5^***N* = 92, **^6^***N* = 92. * These analyses had a convergence problem that was resolved by removing pair ID as random factor, leading to the same results.

**Supplementary Table S11**. fMRI results showing the results of the main contrast (conjunction analysis, and comparison between static decision-making and dynamic decision-making).

| **Main effect of condition** | Cluster | Region | H | **MNI (x,y,z)** | t | k |
| --- | --- | --- | --- | --- | --- | --- |
| **Static decision-making > baseline ∩ Dynamic decision-making > baseline** | 1 | Precuneous Cortex  Frontal Operculum Cortex  IFG, pars triangularis  Middle Temporal Gyrus  Precentral Gyrus  Middle Frontal Gyrus  Temporal Pole  Angular Gyrus  Planum Polare  Lateral Occipital Cortex  Supramarginal Gyrus  Superior Temporal Gyrus | R  R  R  R  R  R  R  R  R  R  R  R | 6 -60 66  42 18 -2  52 14 26  52 -60 8  46 10 30  46 6 40  50 8 -6  60 -46 22  42 -12 -6  44 -66 4  60 -30 42  48 -16 -4 | 12.22  12.13  10.44  10.05  9.98  9.14  9.01  9.00  8.99  8.66  8.49  7.97 | 6883 |
|  | 2 | Superior Frontal Gyrus | R | 4 40 40 | 8.53 | 1013 |
|  | 3 | Frontal Orbital Cortex  Insular Cortex  Planum Polare  Temporal pole  Frontal Operculum Cortex | L  L  L  L  L | -32 24 -6  -42 -12 -6  -46 -2 -10  -48 8 -8  -36 26 10 | 9.17  8.73  6.14  5.73  4.76 | 683 |
|  | 4 | Thalamus | R | 10 -14 10 | 6.68 | 157 |
|  | 5 | Parietal Operculum Cortex | L | -64 -34 20 | 5.86 | 153 |
|  | 6 | Temporal Occipital Fusiform Cortex | R | 46 -60 -20 | 6.99 | 80 |
|  | 7 | Anterior Cingulate Cortex  Posterior Cingulate Cortex | R  R | 2 -4 38  2 -18 46 | 5.56  5.32 | 45 |
|  | 8 | Middle Temporal Gyrus | L | -46 -60 10 | 5.88 | 38 |
|  | 9 | Cerebellum Vermis VIII | L | -6 -62 -34 | 6.01 | 15 |
|  | 10 | Lateral Occipital Cortex | R | 32 -78 36 | 6.01 | 14 |
|  | 11 | Thalamus | R | 6 -24 0 | 5.36 | 9 |
|  | 12 | Planum Temporal | L | -48 -26 0 | 5.64 | 7 |
|  | 13 | Frontal Pole | R | 44 40 28 | 4.94 | 6 |
| **Static decision-making > Dynamic decision-making** | 1 | Heschl´s Gyrus  Planum Temporale | L  L | -46 -24 8  -40 -30 10 | 20.00  18.00 | 1687 |
|  | 2 | Superior Frontal Gyrus  Precentral  Juxtapositional Lobule Cortex | R  R  R | 22 0 56  42 -4 52  8 6 56 | 11.50  7.05  6.42 | 1494 |
|  | 3 | Heschl´s Gyrus  Planum Temporale  Parietal Operculum Cortex | R  R  R | 52 -12 4  64 -18 6  38 -30 18 | 19.50  12.19  10.56 | 1475 |
|  | 4 | Precentral Gyrus | L | -12 -20 42 | 14.20 | 1275 |
|  | 5 | Superior Frontal Gyrus | L | -20 0 60 | 12.20 | 1102 |
|  | 6 | Precentral Gyrus  Lateral Occipital Cortex  Precuneous Cortex | R  R  R | 14 -18 44  14 -18 44  14 -44 50 | 10.60  9.69  7.46 | 1002 |
|  | 7 | Lateral Occipital Cortex | R | 40 -66 4 | 7.96 | 192 |
|  | 8 | Lateral Occipital Cortex | L | -40 -68 6 | 10.40 | 124 |
|  | 9 | Middle Frontal Gyrus | L | -34 38 40 | 7.08 | 122 |
|  | 10 | Putamen | L | -22 18 -8 | 6.76 | 29 |
|  | 11 | Putamen  Caudate | R  R | 24 18 -6  20 22 0 | 6.71  5.93 | 21 |
|  | 12 | IFG, pars triangularis | R | 32 28 10 | 5.84 | 20 |
|  | 13 | Superior Parietal Lobule | L | -30 -42 52 | 5.60 | 20 |
|  | 14 | Caudate | L | -6 14 2 | 5.60 | 16 |
|  | 15 | Caudate | R | 8 16 0 | 5.05 | 14 |
|  | 16 | Precuneous Cortex | R | 20 -60 24 | 5.76 | 14 |
|  | 17 | Middle Frontal Gyrus | R | 30 34 38 | 5.42 | 14 |
|  | 18 | Brain-Stem | R | 6 -34 -10 | 6.15 | 10 |
|  | 19 | Superior Parietal Lobule | R | 28 -44 50 | 5.24 | 10 |
|  | 20 | Brain-Stem | L | -6 -36 -10 | 5.18 | 8 |
| **Dynamic decision-making > Static decision-making** | 1 | Intracalcarine Cortex  Supracalcarine Cortex  Cuneal Cortex  Intracalcarine Cortex  Lingual Gyrus  Occipital Fusiform Gyrus  Inferior Temporal Gyrus  Temporal Fusiform Cortex | R  L  R  L  L L  L  L | 4 -70 10  2 -80 16  6 -80 24  -4 -70 8  -10 -66 2  -20 -80 -14  -50 -54 -16  -34 -42 -20 | 26.17  23.36  23.11  22.29  21.95  17.15  10.67  9.04 | 10888 |
|  | 2 | Temporal Occipital Fusiform Cortex  Posterior Cingulate Cortex  Temporal Occipital Fusiform Cortex  Cerebellum VI  Posterior Cingulate Cortex | L  L  R  L  R | -34 -48 -22  -6 -50 34  28 -40 -20  -28 -46 -22  4 -36 34 | 8.98  8.51  8.47  8.47  7.51 |  |
|  | 3 | Middle Temporal Gyrus  Lateral Occipital Cortex  Superior Parietal Lobule  Superior Temporal Gyrus  Angular Gyrus | L  L  L  L  L | -52 -40 2  -30 -66 46  -30 -58 42  -52 -14 -8  -50 -56 24 | 10.70  9.56  9.15  8.25  7.71 | 1932 |
|  | 4 | IFG, pars triangularis  Middle Frontal Gyrus  IFG, pars opercularis | L  L  L | -50 28 8  -48 20 26  -56 20 18 | 9.39  9.30  7.85 | 1861 |
|  | 5 | Precentral Gyrus  Central Opercular Cortex  Insular Cortex  Putamen  Postcentral Gyrus  Temporal Pole  Supramarginal Gyrus | R  R  R  R  R  R  R | 38 -16 38  38 -12 18  38 -10 10  32 -6 6  46 -28 54  44 16 -12  52 -34 54 | 8.80  8.60  7.78  7.76  5.71  5.32  5.15 | 1550 |
|  | 6 | Lateral Occipital Cortex  Angular Gyrus  Superior Parietal Lobule | R  R  R | 32 -60 36  52 -58 28  44 -48 58 | 9.26  6.35  5.47 | 1155 |
|  | 7 | Frontal Pole  Superior Frontal Gyrus  Frontal Pole | R  L  L | 10 5 36  -6 22 62  -6 58 32 | 7.17  6.61  6.20 | 891 |
|  | 8 | Superior Parietal Lobule | R | 20 -48 72 | 8.59 | 513 |
|  | 9 | Precentral Gyrus  Postcentral Gyrus | L  L | -36 -16 40  -62 -12 28 | 9.28  6.87 | 512 |
|  | 10 | Frontal Pole  Middle Frontal Gyrus | R  R | 40 36 18  50 34 26 | 6.79  6.07 | 347 |
|  | 11 | Middle Temporal Gyrus  Inferior Temporal Gyrus | R  R | 52 -38 -2  48 -48 -12 | 7.36  6.19 | 311 |
|  | 12 | Insular Cortex  Putamen | L  L | -36 -14 18  -30 -6 10 | 7.10  6.04 | 167 |
|  | 13 | Thalamus | R | 24 -30 -2 | 7.72 | 126 |
|  | 14 | Anterior Cingulate Cortex  Anterior Cingulate Cortex  Paracingulate Gyrus | L  R  R | -2 44 10  2 40 12  2 46 -2 | 5.82  5.43  5.13 | 94 |
|  | 15 | Superior Parietal Lobule  Postcentral Gyrus | L  L | -14 -50 74  -16 -44 70 | 5.86  5.82 | 70 |
|  | 16 | Central Opercular Cortex | R | 36 6 14 | 6.49 | 54 |
|  | 17 | Temporal Pole | L | -48 2 -24 | 6.54 | 47 |
|  | 18 | Supramarginal Gyrus | L | -46 -42 42 | 5.28 | 45 |
|  | 19 | Amygdala | L | -26 -6 -18 | 7.98 | 40 |
|  | 20 | Thalamus | L | -22 -30 -2 | 7.99 | 30 |
|  | 21 | Frontal Pole | L | -10 62 18 | 5.20 | 26 |
|  | 22 | Hippocampus/Amygdala | R | 26 -8 -20 | 5.78 | 21 |
|  | 23 | Middle Temporal Gyrus | R | 46 2 -26 | 6.07 | 16 |
|  | 24 | Insular Cortex | L | -38 8 -6 | 5.20 | 12 |
|  | 25 | Middle Frontal Gyrus | R | 40 22 52 | 5.07 | 10 |

*Note*: H = Hemisphere, MNI = Montreal neurological institute, t = t-test statistical value, k = cluster size, L = left, R = right, IFG = inferior frontal gyrus. *All results are significant at *p* < .05 corrected for family-wise error (FWE) at voxel-level (k ≥ 5). Anatomical labeling was performed using the Anatomy toolbox (version 3.0) within the SPM software.

**Supplementary Table S12**. Comparison between cooperation and defection within static and dynamic decision-making phases.

| **Main effect of condition** | Cluster | Region | H | **MNI (x,y,z)** | t | k |
| --- | --- | --- | --- | --- | --- | --- |
| **Static decision-making:**  **cooperation (turn) > defection (continue)** | 1 | Occipital Fusiform Gyrus  Lingual Gyrus  Occipital Fusiform Gyrus  Cerebellum IV  Temporal Occipital Fusiform Cortex  Cerebellum IV  Lateral Occipital Cortex | R  R  L  L  L  R  L | 20 -72 -10  10 -76 -2  -20 -78 -16  -24 -60 -18  -26 -48 -10  24 -54 -18  -22 -84 16 | 11.60  11.10  10.84  10.16  10.10  9.78  9.56 | 9727 |
|  | 2 | Precentral Gyrus  Central Opercular Cortex  Juxtapositional Lobule Cortex  Superior Frontal Gyrus  Postcentral Gyrus | L  L  L  L | -24 -20 74  -50 -20 22  0 -4 56  -12 -8 78  -52 -20 28 | 8.80  8.48  8.41  8.01  7.90 | 7212 |
|  | 3 | Postcentral Gyrus  Supramarginal Gyrus  Parietal Operculum Cortex  Planum Temporale | R  R  R  R | 56 -18 22  66 -26 24  58 -32 28  62 -32 20 | 7.87  7.28  6.49  6.20 | 1428 |
|  | 4 | Central Opercular Cortex  Putamen  Precentral  IFG, pars opercularis | L  L  L  L | -48 4 0  -32 -10 0  -56 6 36  -56 10 20 | 7.11  7.07  6.84  6.75 | 1253 |
|  | 5 | Precentral  Central Opercular Cortex | R  R | 56 8 2  42 4 6 | 7.67  7.64 | 684 |
|  | 6 | Precuneous Cortex  Postcentral Cortex | L  L | -10 -50 64  -16 -46 54 | 6.52  5.37 | 491 |
|  | 7 | Frontal Pole | R | 34 40 32 | 5.91 | 359 |
|  | 8 | Precuneous Cortex | R | 8 -58 64 | 6.55 | 308 |
|  | 9 | Posterior Cingulate Cortex  Precuneous Cortex | R  R | 14 -26 38  14 -36 44 | 6.15  5.90 | 209 |
|  | 10 | Caudate | L | -18 -14 20 | 5.73 | 69 |
|  | 11 | Thalamus | L | -10 -20 4 | 5.61 | 66 |
|  | 12 | Superior Frontal Gyrus  Middle Frontal Gyrus | R  R | 24 10 54  30 16 54 | 5.29  5.09 | 66 |
|  | 13 | Precentral Gyrus | R | 30 -12 58 | 5.58 | 43 |
|  | 14 | Lateral Occipital Cortex | R | 20 -70 52 | 5.05 | 32 |
|  | 15 | Thalamus | L | -18 -32 12 | 5.35 | 15 |
| **Static decision-making:**  **defection (continue) > cooperation (turn)** | 1 | Anterior Cingulate Cortex | R | 4 44 0 | 5.09 | 12 |
| **Dynamic decision-making:**  **cooperation (turn) > defection (continue)** | 1 | Lingual Gyrus  Intracalcarine Cortex | R  R | 14 -78 -2  10 -86 16 | 6.78  5.42 | 218 |
|  | 2 | Frontal Pole | R | 50 36 26 | 5.33 | 19 |
| **Dynamic decision-making:**  **defection (continue) > cooperation (turn)** | 1 | Paracingulate Gyrus  Juxtapositional Lobule Cortex  Superior Frontal Gyrus  Anterior Cingulate Cortex | L  R  R  R | -6 16 38  6 0 62  6 2 72  4 44 10 | 7.79  6.92  6.46  5.00 | 2303 |
|  | 2 | Precentral Cortex  Postcentral Cortex | L  L | -40 -14 54  -50 -16 42 | 6.55  5.73 | 509 |
|  | 3 | Frontal Pole  Middle Frontal Gyrus | L  L | -30 38 42  -28 36 28 | 5.96  5.39 | 405 |
|  | 4 | Precentral Gyrus  Central Opercular Cortex  Frontal Opercular Cortex | L  L  L | -56 2 18  -34 8 12  -44 10 6 | 6.04  5.52  5.40 | 183 |
|  | 5 | Precentral Gyrus | R | 50 -8 42 | 5.89 | 99 |
|  | 6 | Frontal Pole | L | -4 56 16 | 5.40 | 94 |
|  | 7 | Frontal Pole | R | 4 66 4 | 5.53 | 48 |
|  | 8 | Parietal Operculum Cortex  Central Opercular Cortex | L  L | -44 -24 20  -50 -20 22 | 5.05  4.89 | 40 |
|  | 9 | Precentral Gyrus | R | 60 4 18 | 5.35 | 26 |
|  | 10 | Superior Frontal Gyrus | L | -16 12 66 | 5.34 | 21 |
|  | 11 | Putamen | R | 20 16 4 | 5.29 | 18 |
|  | 12 | Central Opercular Cortex | R | 52 6 2 | 5.03 | 15 |
|  | 13 | Precentral Gyrus | R | 42 -14 36 | 4.87 | 12 |
|  | 14 | Amygdala | R | 28 -10 -14 | 5.19 | 8 |
|  | 15 | Frontal Pole | R | 6 58 22 | 4.87 | 7 |
|  | 16 | Thalamus | L | -16 -24 2 | 4.92 | 5 |
|  | 17 | Posterior Cingulate Cortex | L | -4 -26 46 | 4.87 | 5 |

*Note*: H = Hemisphere, MNI = Montreal neurological institute, t = t-test statistical value, k = cluster size, L = left, R = right, IFG = inferior frontal gyrus. * All results are significant at *p* < .05 corrected for family-wise error (FWE) at voxel-level (k ≥ 5). Anatomical labeling was performed using the Anatomy toolbox (version 3.0) within the SPM software.

**Supplementary Table S13.** ROI findings applying the mentalizing mask (Neurosynth).

| **Main effect of condition** | Cluster | Region | H | **MNI (x,y,z)** | t | k |
| --- | --- | --- | --- | --- | --- | --- |
| **[Static decision-making > baseline ∩ Dynamic decision-making > baseline] ^ mentalizing mask** | 1 | Lateral Occipital Cortex  Angular Gyrus  Middle Temporal Gyrus  Supramarginal Gyrus | R  R  R  R | 52 -62 10  48 -46 16  58 -46 20  48 -42 16 | 10.04  8.50  8.31  6.26 | 379 |
|  | 2 | IFG, pars opercularis  Precentral | R  R | 52 14 28  46 10 30 | 10.18  9.98 | 342 |
|  | 3 | Insular Cortex | R | 40 20 -2 | 11.90 | 193 |
|  | 4 | Superior Frontal Gyrus | R | 4 30 44 | 7.72 | 48 |
|  | 5 | Insular Cortex  Frontal Orbital Cortex | L  L | -30 26 -2  -42 20 -6 | 7.74  6.16 | 31 |
|  | 6 | Middle Frontal Gyrus | R | 48 12 40 | 7.13 | 26 |
|  | 7 | Precuneous Cortex | R | 4 -54 48 | 5.90 | 24 |
|  | 8 | Lateral Occipital Cortex | R | 20 62 64 | 5.83 | 23 |
|  | 9 | Middle Temporal Gyrus | L | -46 60 10 | 5.88 | 17 |
|  | 10 | Frontal Orbital Cortex | L | -32 22 -10 | 8.30 | 15 |
|  | 11 | Superior Parietal Lobule | R | 36 -44 52 | 6.08 | 14 |
|  | 12 | Superior Frontal Gyrus | R | 4 50 30 | 4.98 | 12 |
|  | 13 | Superior Temporal Gyrus | R | 52 -24 -4 | 6.03 | 8 |
|  | 14 | Superior Frontal Gyrus | R | 4 40 36 | 7.41 | 7 |
|  | 15 | Thalamus | R | 8 -6 8 | 6.26 | 6 |
|  | 16 | Supramarginal Gyrus | R | 62 -42 20 | 7.50 | 6 |
|  | 17 | Thalamus | R | 6 -24 0 | 5.36 | 5 |
|  | 18 | Thalamus | R | 8 -12 6 | 5.71 | 5 |
| **[Static decision-making > Dynamic decision-making] ^ mentalizing mask** | 1 | Lateral Occipital Cortex | R | 44 -62 6 | 6.67 | 23 |
|  | 2 | Precentral Gyrus | L | -12 -22 42 | 13.60 | 20 |
|  | 3 | Paracingulate Gyrus | R | 8 10 50 | 6.10 | 19 |
|  | 4 | Lateral Occipital Cortex | R | 20 -62 58 | 7.21 | 11 |
|  | 5 | Lateral Occipital Cortex | L | -42 -70 4 | 10.20 | 5 |
| **[Dynamic decision-making > Static decision-making] ^ mentalizing mask** | 1 | Precuneous Cortex  Precuneous Cortex  Posterior Cinculate Gyrus  Posterior Cinculate Gyrus | L  R  L  R | -2 -66 18  2 -66 22  -6 -50 34  4 -48 28 | 13.16  9.74  8.51  6.07 | 579 |
|  | 2 | IFG, pars triangularis  Middle Frontal Gyrus  Frontal Operculum Cortex | L  L  L | -50 28 8  -48 20 26  -46 26 -2 | 9.39  9.30  7.31 | 494 |
|  | 3 | Superior Parietal Lobule  Lateral Occipital Cortex  Angular Gyrus  Middle Temporal Gyrus | L  L  L  L | -32 -58 44  -44 -66 30  -50 -56 24  -58 -48 8 | 8.43  8.24  7.85  6.45 | 344 |
|  | 4 | Frontal Pole  Frontal Pole | R  L | 10 58 32  -6 58 32 | 6.69  6.20 | 274 |
|  | 5 | Middle Temporal Gyrus  Superior Temporal Gyrus | L  L | -52 -40 2  -54 -14 -8 | 10.70  8.03 | 131 |
|  | 6 | Lateral Occipital Cortex  Angular Gyrus | R  R | 50 -60 30  44 -54 24 | 6.42  5.32 | 97 |
|  | 7 | Inferior Temporal Gyrus | L | -48 -58 -12 | 10.60 | 26 |
|  | 8 | Superior Frontal Gyrus | L | -6 18 62 | 5.84 | 21 |
|  | 9 | Lateral Occipital Cortex | R | 46 -62 44 | 7.01 | 20 |
|  | 10 | Frontal Pole | L | -10 60 20 | 5.19 | 16 |
|  | 11 | Precuneous Cortex | R | 18 -60 12 | 11.00 | 10 |
|  | 12 | Amygdala | R | 26 -8 -18 | 5.53 | 9 |
|  | 13 | Middle Frontal Gyrus | R | 46 32 20 | 5.86 | 9 |
|  | 14 | Amygdala | L | -24 -6 -18 | 7.54 | 7 |
|  | 15 | Middle Temporal Gyrus | R | 50 -30 -2 | 5.83 | 7 |
|  | 16 | Middle Frontal Gyrus | R | 50 30 28 | 5.31 | 7 |
|  | 17 | Intracalcarine Gyrus | R | 14 -80 8 | 15.00 | 6 |
|  | 18 | Anterior Cingulate Gyrus | R | 2 38 12 | 5.32 | 6 |

*Note*: H = Hemisphere, MNI = Montreal neurological institute, t = t-test statistical value, k = cluster size, L = left, R = right, IFG = inferior frontal gyrus. * All results are significant at *p* < .05 corrected for family-wise error (FWE) at voxel-level (k ≥ 5). Anatomical labeling was performed using the Anatomy toolbox (version 3.0) within the SPM software.


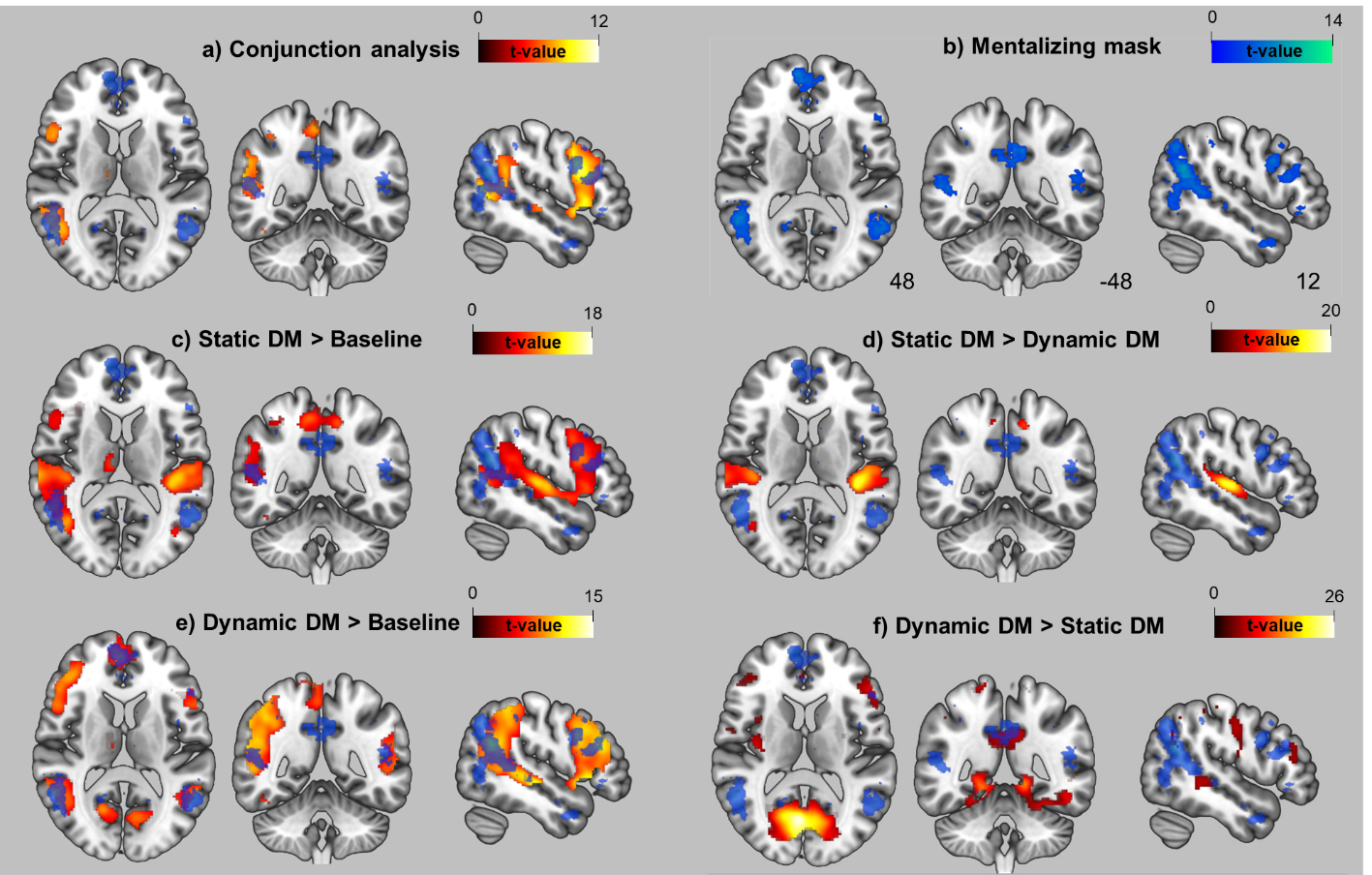


**Figure S4**. a) Conjunction analysis results representing the shared activation between static decision-making and dynamic decision-making (represented in orange) and showing considerable overlap with the mentalizing mask (represented in blue). b) mentalizing mask from the automated Neurosynth meta-analysis of mentalizing (<https://neurosynth.org/analyses/terms/mentalizing/>). c) Static decision-making over baseline (represented in orange) showing partial overlap with the mentalizing mask (represented in blue). d) Static decision-making over dynamic decision-making (represented in orange) showing limited overlap with the mentalizing mask (represented in blue). e) Dynamic decision-making over baseline (represented in orange) showing considerable overlap with the mentalizing mask (represented in blue), being higher than static decision-making over baseline contrast. f) Dynamic decision-making over static decision-making (represented in orange) showing partial overlap with the mentalizing mask (represented in blue). The statistical threshold maps are set to *p* < .05, FWE corrected at voxel level. The figure was created with MRIcroGL1.2. software, and the coordinate values corresponding to the standard 2D slice coordinate system of the software (x = 48, y = -48, z = 12) for all figures (a, b, c, d, e, f). *Note*: DM = decision-making.

**Supplementary Table S14.** Exploratory whole brain sex differences per contrasts.

| **Main effect of condition** | Cluster | Region | H | **MNI (x,y,z)** | t | k |
| --- | --- | --- | --- | --- | --- | --- |
| **[Static DM cooperation > defection] men > women*** |  | No cluster survived FWE-correction |  |  |  |  |
| **[Static DM cooperation > defection] men > women**** | 1 | Superior Frontal Gyrus  Juxtapositional Lobule Cortex | R  R | 18 4 72  0 8 62 | 3.99  3.94 | 128 |
| **[Static DM defection > cooperation] men > women*** | 1  2  3  4  5  6  7 | Supramarginal gyrus  Temporal Occipital Fusiform Cortex  Temporal Occipital Fusiform Cortex  Putamen  Lingual gyrus  Temporal Occipital Fusiform Cortex  Parietal operculum cortex | R  R  R  L  L  L  L | 62 -30 26  34 -50 14  30 -56 -12  -32 -16 -4  -20 -46 -8  -32 -54 -6  -56 -36 28 | 5.57  5.35  5.25  5.16  5.13  5.04  5.1 | 12  7  5  2  1  1  1 |
| **[Static DM defection > cooperation] men > women**** | 1 | Temporal Occipital Fusiform Cortex  Cerebellum VI  Occipital Fusiform | R  R  R | 34 -50 -14  30 -60 -22  38 -66 -16 | 5.35  4.33  3.71 | 701 |
|  | 2 | Lingual Gyrus  Temporal Occipital Fusiform Cortex  Cerebellum VI  Thalamus | L  L  L  L | -20 -46 -8  -32 -54 -6  -24 -68 -20  -14 -38 -2 | 5.13  5.04  4.24  3.36 | 688 |
|  | 3 | Parietal Operculum Cortex  Supramarginal Gyrus | L  L | -56 -36 28  -60 -32 30 | 5.10  4.83 | 517 |
|  | 4 | Cuneal Cortex  Lateral Occipital Cortex | L  L | -10 -76 24  -24 -86 20 | 4.79  4.39 | 389 |
|  | 5 | Hippocampus  Lingual Gyrus  Precuneous Cortex | R  R  R | 16 -40 4  26 -46 2  14 -52 6 | 4.34  4.26  3.88 | 223 |
|  | 6 | Supramarginal Gyrus  Parietal Operculum Cortex | R  R | 62 -30 26  52 -30 30 | 5.57  3.46 | 215 |
|  | 7 | Precuneous Cortex | L | -14 -56 34 | 4.67 | 177 |
| **[Dynamic DM cooperation > defection] men > women*** |  | No cluster survived FWE-correction |  |  |  |  |
| **[Dynamic DM cooperation > defection] men > women**** |  | No cluster survived FWE-correction |  |  |  |  |
| **[Dynamic DM defection > cooperation] men > women*** | 1  2  3 | Precuneous Cortex  Lateral Occipital Cortex  Supramarginal Gyrus | R  R  L | 10 -48 56  16 -62 66  -62 -32 26 | 5.42  5.21  5.21 | 6  2  1 |
| **[Dynamic DM defection > cooperation] men > women**** | 1 | Precuneous Cortex  Lateral Occipital Cortex  Precuneous Cortex | R  R  L | 10 -48 56  16 -62 66  -10 -50 56 | 5.42  5.21  4.82 | 912 |
|  | 2 | Supramarginal Gyrus | L | -62 -32 26 | 5.21 | 192 |

*Note*: H = Hemisphere, DM = decision-making, MNI = Montreal neurological institute, t = t-test statistical value, k = cluster size, L = left, R = right. * Results are significant at p < .05 at voxel-level (FWE-correction), (k ≥ 5). **Results are significant at cluster-defining threshold of *p* < .001 with family-wise error correction (FWE-corrected *p* < .05) on a cluster level. No significant results for reverse contrast (women > men). Anatomical labeling was performed using the Anatomy toolbox (version 3.0) within the SPM software.


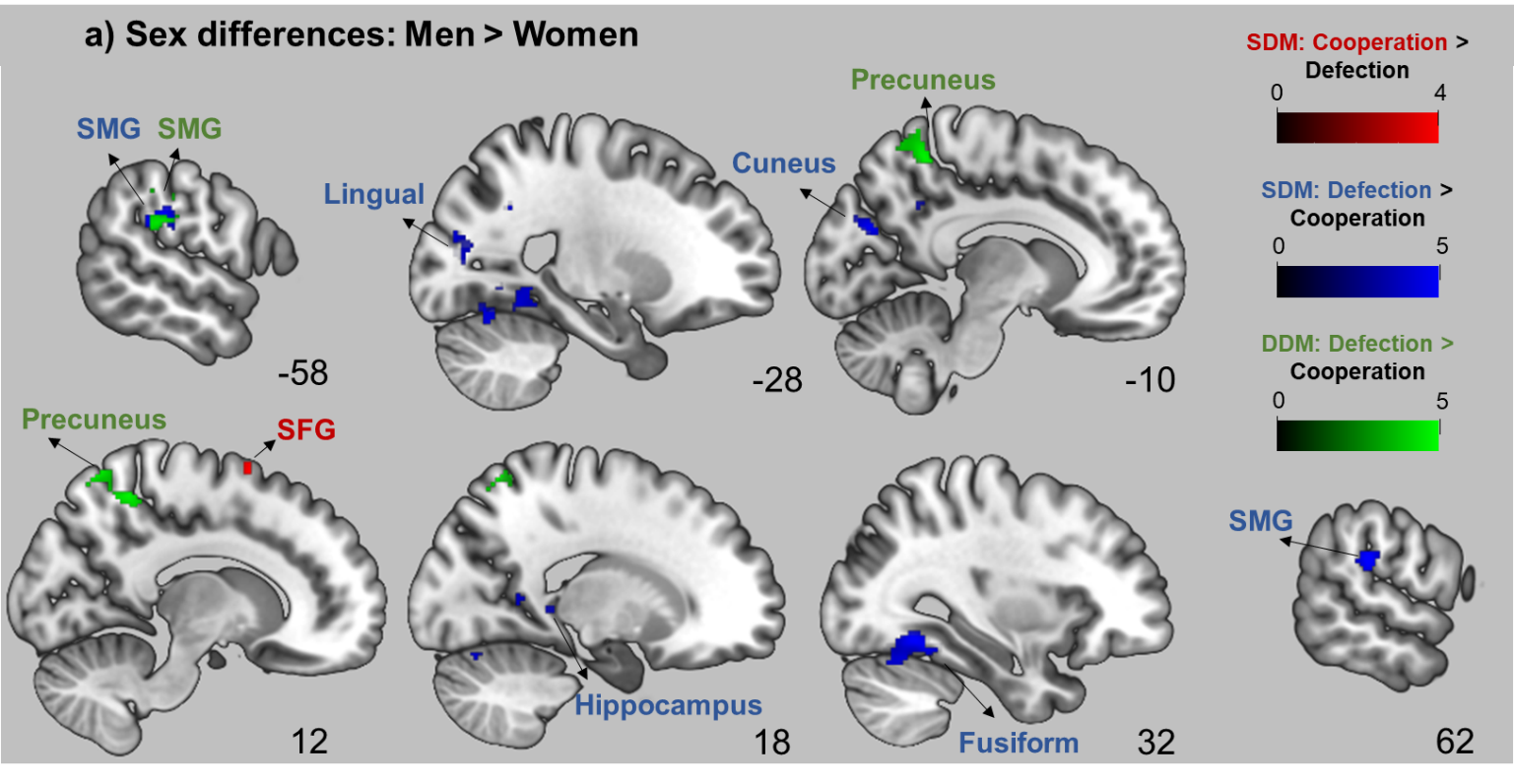


**Figure S5**. Figure representing whole brain sex differences. Independent t-tests for each contrast (static decision-making cooperation > defection, static decision-making defection > cooperation, dynamic decision-making cooperation > defection, and dynamic decision-making defection > cooperation) were calculated using a threshold map set cluster-defining threshold of *p* < .001 with family-wise error correction (FWEc: *p* < .05) on a cluster level. Dynamic decision-making cooperation > defection did not show any significant results. Only men > women contrast yielded significant results. The figure was created with MRIcroGL1.2. software, and the coordinate values corresponding to the standard 2D slice coordinate system of the software. *Note*: SDM = static decision-making, DDM = dynamic decision-making, SMG = supramarginal gyrus, SFG = superior frontal gyrus.

**References**

Achtziger, A., Alós-Ferrer, C., Hügelschäfer, S., & Steinhauser, M. (2014). The neural basis of belief updating and rational decision making. *Social Cognitive and Affective Neuroscience*, *9*(1), 55–62. https://doi.org/10.1093/scan/nss099

Anderson, C., John, O. P., & Keltner, D. (2012). The Personal Sense of Power: The Personal Sense of Power. *Journal of Personality*, *80*(2), 313–344. https://doi.org/10.1111/j.1467-6494.2011.00734.x

Buss, A. H., & Perry, M. (1992). The Aggression Questionnaire. *Journal of Personality and Social Psychology*, *63*(3), 452–459. https://doi.org/10.1037/0022-3514.63.3.452

Christie, R., & Geis, F. (1970). *Studies in Machiavellianism*. Academic Press.

Hernandez-Pena, L., Hoppe, W., Koch, J., Keeler, C., Waller, R., Habel, U., Sijben, R., & Wagels, L. (2023). The role of dominance in sibling relationships: Differences in interactive cooperative and competitive behavior. *Scientific Reports*, *13*(1), 11863. https://doi.org/10.1038/s41598-023-38936-7

Kim, H.-Y. (2017). Statistical notes for clinical researchers: Chi-squared test and Fisher’s exact test. *Restorative Dentistry & Endodontics*, *42*(2), 152. https://doi.org/10.5395/rde.2017.42.2.152

Kümmerli, R., Colliard, C., Fiechter, N., Petitpierre, B., Russier, F., & Keller, L. (2007). Human cooperation in social dilemmas: Comparing the Snowdrift game with the Prisoner’s Dilemma. *Proceedings of the Royal Society B: Biological Sciences*, *274*(1628), 2965–2970. https://doi.org/10.1098/rspb.2007.0793

Normann, H.-T., & Wallace, B. (2012). The impact of the termination rule on cooperation in a prisoner’s dilemma experiment. *International Journal of Game Theory*, *41*(3), 707–718. https://doi.org/10.1007/s00182-012-0341-y

Ortmann, A., & Tichy, L. K. (1999). Gender differences in the laboratory: Evidence from prisoner’s dilemma games. *Journal of Economic Behavior & Organization*, *39*(3), 327–339. https://doi.org/10.1016/S0167-2681(99)00038-4

Patton, J. H., Stanford, M. S., & Barratt, E. S. (1995). Factor structure of the barratt impulsiveness scale. *Journal of Clinical Psychology*, *51*(6), 768–774. https://doi.org/10.1002/1097-4679(199511)51:6<768::AID-JCLP2270510607>3.0.CO;2-1

Ryckman, R. M., Hammer, M., Kaczor, L. M., & Gold, J. A. (1990). Construction of a Hypercompetitive Attitude Scale. *Journal of Personality Assessment*, *55*(3–4), 630–639. https://doi.org/10.1080/00223891.1990.9674097

Ryckman, R. M., Hammer, M., Kaczor, L. M., & Gold, J. A. (1996). Construction of a Personal Development Competitive Attitude Scale. *Journal of Personality Assessment*, *66*(2), 374–385. https://doi.org/10.1207/s15327752jpa6602_15

Suessenbach, F., Loughnan, S., Schönbrodt, F. D., & Moore, A. B. (2019). The Dominance, Prestige, and Leadership Account of Social Power Motives. *European Journal of Personality*, *33*(1), 7–33. https://doi.org/10.1002/per.2184

Zuroff, D. C., Fournier, M. A., Patall, E. A., & Leybman, M. J. (2010). Steps toward an evolutionary personality psychology: Individual differences in the social rank domain. *Canadian Psychology / Psychologie Canadienne*, *51*(1), 58–66. https://doi.org/10.1037/a0018472
